# Synthesis of the Core Framework of the Cornexistins by Intramolecular Nozaki-Hiyama-Kishi Coupling

**DOI:** 10.3390/molecules24142654

**Published:** 2019-07-22

**Authors:** Anthony Aimon, Louis J. Farrugia, J. Stephen Clark

**Affiliations:** School of Chemistry, Joseph Black Building, University of Glasgow, University Avenue, Glasgow, G12 8QQ, UK

**Keywords:** cornexistins, nonadride, natural product, herbicide, nine-membered carbocycle, Nozaki-Hiyama-Kishi reaction, cyclisation

## Abstract

A new and direct approach to the construction of the core framework of the herbicidal natural products cornexistin and hydroxycornexistin has been developed. Formation of the nine-membered carbocycle found in the natural products has been accomplished by an intramolecular Nozaki-Hiyama-Kishi reaction between a vinylic iodide and an aldehyde. Good yields of carbocyclic products were obtained from the reaction, but diastereomeric mixtures of allylic alcohols were produced. The cyclisation reaction was successful irrespective of the relative configuration of the stereogenic centres in the cyclisation precursor.

## 1. Introduction

### 1.1. Isolation and Herbicial Activities of the Cornexistins

**Figure 1:**
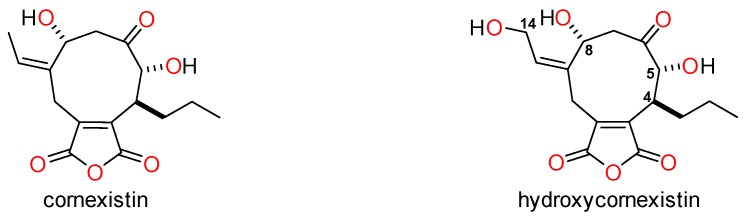
The cornexistin natural products.

Cornexistin and hydroxycornexistin are structurally unique members of the nonadride family of natural products (Figure 1) [1,2,3,4,5]. Cornexistin was first isolated from a culture of the fungus *Paecilomyces variotii* Bainier (strain SANK 21086) by researchers at Sankyo Co. in 1987 and hydroxycornexistin was isolated from the same strain of the fungus by workers at DowElanco eight years later. Both compounds have attracted the interest of the agrochemical industry because of the potent and selective herbicidal activities they possess and because they are prone to rapid degradation on exposure to light or when treated with acid or base [6]. The cornexistins are unusual members of the nonadride family of natural products because they possess a single maleic anhydride unit embedded in their structures whereas most of the other nonadrides possess two [7].

Cornexistin and hydroxycornexistin both display potent herbicidal activity against grasses and broadleaf weeds but are well tolerated by some crop plants [1−5]. Hydroxycornexistin has particularly high activity and is effective against many types of broadleaf weeds [4,5]. It has been suggested that the cornexistins, or metabolised products thereof, exert their herbicidal activities by interfering with one or more aspartate amino transferase enzymes [8], but their precise mode of action has yet to be elucidated fully. The selective and potent herbicidal activity of the cornexistins combined with their apparently novel mode of action and non-persistence in the environment means that these natural products have significant potential as lead compounds in the search for novel, biorational post-emergence weed control agents that can be used in arable crop production.

### 1.2. Previous Synthetic Studies on the Cornexistins

Although the cornexistins have significant potential as novel lead compounds in the search for new herbicides, they have been the subject of few synthetic studies and very little has been published concerning their total synthesis. Aside from our own previously published work (vide infra) [9,10], the only attempt to synthesise either cornexistin or hydroxycornexistin to have been reported is that of Taylor and co-workers [11]. In this study, the oxidative ring opening of a hexahydroindene system was used to create the fully functionalised nine-membered carbocyclic core structure. However, this approach did not culminate in the total synthesis of either natural product.

Our interest in both cornexistin and hydroxycornexistin as targets for total synthesis was aroused by the combination of the synthetic challenges presented by the natural products and their potent herbicidal activities. Both compounds possess a relatively rare nine-membered carbocycle fused to a highly reactive maleic anhydride unit, a combination of structural features that poses a formidable test to conventional methods of ring construction. The cornexistins also present several interesting problems concerning stereocontrol; control of the exocyclic alkene configuration is especially challenging.

We have already shown that a ring-closing metathesis (RCM) reaction can be used to construct the core of the cornexistins from a relatively simple diene precursor [9]. Dihydroxylation of the resulting Δ^5,6^-cyclononene resulted in installation of hydroxyl groups at C-5 and C-6. Unfortunately, the configuration at the hydroxyl-bearing carbon (C-5) was not that found in the natural products and all attempts to invert the configuration at this stereogenic centre were unsuccessful. Ultimately, a synthesis of 5-*epi*-hydroxycornexistin was completed instead of the natural product [10].

The failure of the original route motivated us to explore a completely new approach to the synthesis of cornexistin and hydroxycornexistin. In this second-generation approach, we planned to create the C-5 stereogenic centre with the correct configuration during the cyclisation reaction used to assemble the nine-membered ring, instead of attempting to perform late-stage introduction of the hydroxyl group after ring construction.

### 1.3. Retrosynthetic Analysis of the Cornexistins and Synthesis Design

**Scheme 1:**
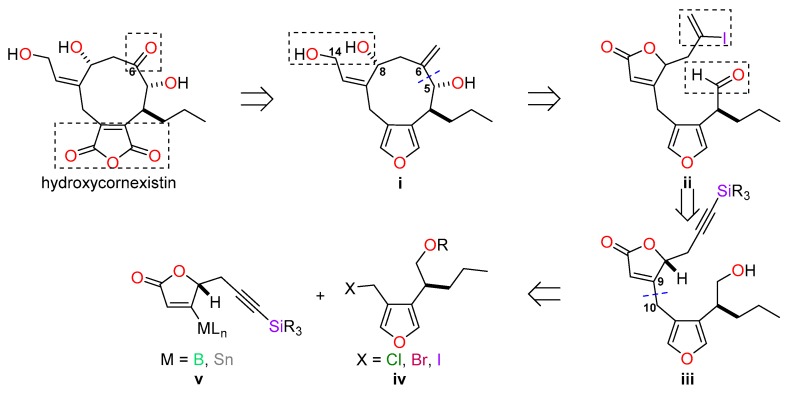
Retrosynthetic analysis of hydroxycornexistin.

The synthetic strategy we chose to adopt in our second-generation approach to the synthesis of the cornexistins was informed by the retrosynthetic analysis presented in Scheme 1. Conversion of the C-6 carbonyl group into an exocyclic alkene and transformation of the maleic anhydride unit into a furan suggested i as a late-stage intermediate. Tethering of the C-8 hydroxyl group and C-14 carbon in the form of a butenolide and ring-opening of the C-5–C-6 bond by scission of the allylic alcohol then led to the aldehyde ii. Subsequent functional group conversion of the iodoalkene into an alkyne and the aldehyde into a hydroxyl group suggested the alcohol iii as intermediate. Finally, cleavage of the C-9–C-10 bond resulted in the disconnection of the compound into two relatively simple fragments of similar size and complexity: the halomethylfuran iv and the metallated butenolide v.

The key step in the synthetic route suggested by the retrosynthetic analysis presented in Scheme 1 was the intramolecular Nozaki-Hiyama-Kishi (NHK) reaction of a vinylic halide with an aldehyde [12]. At the outset of our synthetic studies there appeared to be no published examples of the use of this reaction to construct a nine-membered carbocycle. However, after completion of the work described in this paper [13], Hosokawa and co-workers reported the use of an intramolecular NHK reaction to prepare a nine-membered carbocycle during their synthesis of the fused bicyclic core structure of the marine natural product cristaxenicin A [14].

## 2. Results and Discussion

### 2.1. Synthesis of the Butenolide Fragment 4

**Scheme 2:**
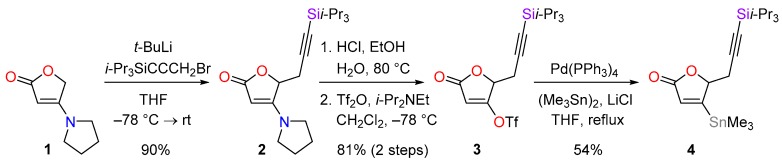
Synthesis of the stannylated crotonolactone **4**.

Synthetic work commenced with the four-step synthesis of the stannylated lactone **4** (corresponding to intermediate **v** in the retrosynthetic analysis) from the simple 4-aminobut-2-enolide **1**, which was prepared by the condensation reaction of tetronic acid with pyrrolidine (Scheme 2) [15]. Deprotonation of the lactone **1** with *t*-butyl lithium and alkylation of the resulting anion with TIPS-protected propargyl bromide [16] afforded the alkyne **2** in high yield when an excess (typically five equivalents) of the alkylating agent was employed. Enamine hydrolysis under acidic conditions and conversion of the resulting enol into the stable enol triflate **3** was followed by a palladium-catalysed reaction with hexamethyl ditin to produce the required stannane **4** (NMR spectra in Supplementary Material) in reasonable yield [17].

### 2.2. Synthesis of the Chloromethylfuran Fragment ***12***

**Scheme 3:**
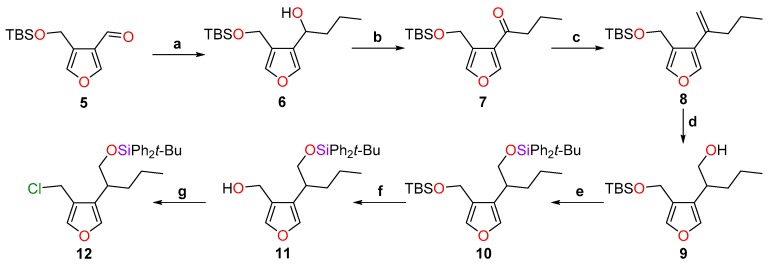
Synthesis of the chloromethylfuran **12**. Reagents and conditions: **a** *n*-PrMgCl, THF, –78 °C→rt, 90%; **b** MnO_2_, CH_2_Cl_2_, reflux, 90%; **c** Ph_3_PCH_3_Br, NaHMDS, THF, 0 °C, 95%; **d** (i) 9-BBN, THF, 55 °C, (ii) NaOH aq., EtOH, 0 °C, (iii) 30% H_2_O_2_, 50 °C, 89%; **e** *t*-BuPh_2_SiCl, Et_3_N, DMAP, CH_2_Cl_2_, rt, 91%; **f** PPTS, EtOH, 40 °C, 94%; **g** MeSO_2_Cl, Et_3_N, CH_2_Cl_2_, rt, 90%.

Chloride **12** (corresponding to **iv** in the retrosynthetic analysis), the coupling partner required for construction of the complete carbon skeleton of hydroxycornexistin, was synthesised from the known aldehyde **5** (available from a 3,4-furandicarboxylate diester) as shown in Scheme 3 [18]. Grignard addition of *n*-propylmagnesium bromide to the aldehyde **5** delivered the alcohol **6** and this compound was oxidised with manganese (IV) oxide to produce the ketone **7**. Wittig methylenation of the ketone **7** afforded the alkene **8** and a subsequent hydroboration reaction provided the alcohol **9**. Protection of the alcohol as a *t*-butyldiphenylsilyl ether produced the furan **10** and selective cleavage of the *t*-butyldimethylsilyl ether delivered the alcohol **11**. The alcohol **11** was converted into the chloride **12** (NMR spectra in Supplementary Material) directly and in high yield upon treatment with methanesulfonyl chloride [19]. Thus, the aldehyde **5** was converted into the chloride **12** in 7 steps and with an overall yield of 50% (an average yield of > 90% per step). 

### 2.3. Palladium-Mediated Coupling of the Stannane and Chloride to Complete the Skeleton of Hydroxycornexistin

**Scheme 4:**
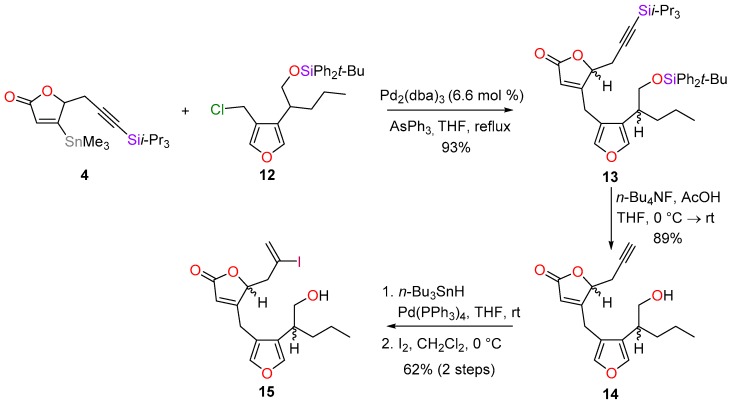
Assembly of the complete carbon backbone by Stille coupling of the vinylic stannane **4** and the chloride **12**.

The stannane **4** and the chloride **12** were subjected to a high-yielding sp^2^–sp^3^ coupling reaction mediated by the combination of tris(dibenzylideneacetone)dipalladium(0) and triphenylarsine (Scheme 4) [20]. The resulting bicyclic compound **13** was then subjected to double desilylation to reveal the alcohol **14**. Palladium-mediated regioselective hydrostannylation of the alkyne followed by tin-iodine exchange delivered the vinylic iodide **15** [21]. A mixture of diastereomers had been generated as a consequence of the coupling of two racemic fragments (**4** and **12**), but the diastereomeric iodides **15a** and **15b** (NMR spectra in Supplementary Materials) were separable by standard silica gel column chromatography, which allowed them to be characterized fully (vide infra) and the NHK cyclisation reaction of each isomer to be explored separately.

### 2.4. Construction of the Nine-Membered Ring of Hydroxycornexistin by Use of the Nozaki-Hiyama-Kishi Reaction

**Scheme 5:**
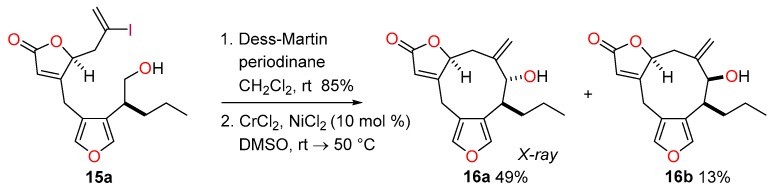
Cyclisation of **15a** to give the nine-membered ring by use of a Nozaki-Hiyama-Kishi reaction.

Cyclisation of the aldehyde (corresponding to **ii** in Scheme 1) generated from the vinylic iodide **15a**, the diastereomer that possesses incorrect relative stereochemistry (S*,S*), was explored first (Scheme 5). Oxidation was performed using the Dess-Martin periodinane and treatment of the resulting aldehyde with a large excess of chromium(II) chloride and a sub-stoichiometric amount (10 mol%) of nickel(II) chloride in dry, degassed DMSO promoted an intramolecular NHK reaction to give the tricyclic products **16a** and **16b** in yields of 49% and 13% respectively (NMR spectra in Supplementary Material). The major isomer (**16a**) was a crystalline solid and crystals suitable for analysis by X-ray diffraction were obtained [22]. X-ray diffraction data confirmed that the alcohol **16a** has the relative stereochemistry shown and by extension that **15a** is the diastereomer shown in Scheme 5. 

**Scheme 6:**
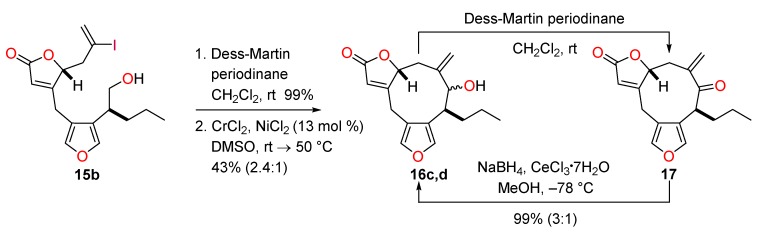
Cyclisation of **15a** to give the nine-membered ring by use of a Nozaki-Hiyama-Kishi reaction and an attempt to improve the diastereomeric ratio by sequential alcohol oxidation and ketone reduction.

The cyclization reaction of the aldehyde derived from the alcohol **15b** was also explored (Scheme 6). Oxidation of the alcohol **15b** proceeded in excellent yield and treatment of the resulting aldehyde with a large excess of chromium(II) chloride and a sub-stoichiometric amount (13 mol%) of nickel(II) chloride in dry, degassed DMSO produced an inseparable mixture (2.4:1 ratio, C-5 configuration not established) of the diastereomeric alcohols **16c** and **16d** (NMR spectra in Supplementary Material) in 43% yield. In an attempt to improve the diastereomeric ratio of the allylic alcohols, an oxidation and reduction sequence was performed. Oxidation of the mixture of alcohols produced the enone **17** (^1^H-NMR spectrum in Supplementary Material) and subsequent Luche reduction returned a mixture of the alcohols **16c** and **16d** with a similar diastereomeric ratio to that obtained from the original NHK cyclization reaction.

## 3. Conclusions

We have shown that it is possible to assemble the core structure of the cornexistins by a short and convergent route. Palladium-mediated sp^2^–sp^3^ coupling of the vinylic stannane **4** with the chloromethylfuran **12**, conversion of the coupled product **13** into the vinylic iodides **15** and subsequent sequential oxidation and intramolecular Nozaki-Hiyama-Kishi (NHK) reaction was used to construct the nine-membered ring. The intramolecular NHK reactions of aldehydes derived from alcohols **15a** and **15b** have been accomplished in reasonable yield, which demonstrates that cyclisation is successful irrespective of the relative configuration of the stereogenic centres present in the vinylic iodide. This finding means that either diastereomer can be used as a cyclisation precursor, which should permit greater flexibility in latter stages of the synthetic route.

## 4. Materials and Methods 

Air and/or moisture sensitive reactions were performed under an atmosphere of Argon in flame-dried apparatus. When necessary, solvents were dried and purified using a Pure Solv™ solvent purification system (SPS). IR spectra were recorded using a type IIa diamond single reflection element on a Shimadzu FTIR-8400 instrument. The IR spectrum of the compound (solid or liquid) was obtained by analysis of a thin layer at ambient temperature. ^1^H and ^13^C-NMR spectra were recorded using either a Bruker 400 MHz or 500 MHz Spectrospin spectrometer at ambient temperature; ^13^C-NMR NMR spectra were recorded at 101 MHz or 126 MHz. Mass spectra were obtained by ionisation under EI, FAB, CI and ESI conditions on a Jeol MStation JMS-700 instrument. Elemental analyses were performed on an Exeter Analytical Elemental Analyser EA 440 by technical staff at the University of Glasgow. Melting points were recorded with an Electrothermal IA 9100 apparatus.

### 4.1. Synthesis of 4-(pyrrolidin-1-yl)-5-(3-triisopropylsilylprop-2-ynyl)furan-2(5H)-one *(**2**)*

To a solution of **1** (653 mg, 4.26 mmol) in THF (6 mL) at −78 °C was added carefully a solution of t-BuLi (4.0 mL of a 1.6 m solution in hexane, 6.4 mmol, 1.5 equiv.). The mixture was stirred for 30 min before a solution of 3-bromo-1-(triisopropylsilyl)-1-propyne (5.88 g, 21.4 mmol, 5.0 equiv.) in THF (8 mL) cooled to −78 °C was added carefully. After 3 h, the mixture was allowed to warm to room temperature over 45 min, and the reaction was quenched by the addition of saturated aqueous NH_4_Cl solution (10 mL). The mixture was diluted with ethyl acetate (20 mL) and the phases were separated. The aqueous phase was extracted with ethyl acetate (2 × 10 mL) and the combined organic extracts were washed with brine (20 mL), then dried over MgSO_4_, filtered and concentrated in vacuo. The crude material was purified by flash column chromatography (PE-EtOAc, 7:3) to give the desired alkyne **2** (1.34 g, 90%) as a colourless solid. M.p. 122−125 °C; R_f_ = 0.23 (PE-EtOAc, 1:1); IR ν_max_ 2942, 2928, 2888, 2865, 2180, 1722, 1610, 994, 920, 901, 882, 853, 839, 773 cm^−1^; ^1^H-NMR (400 MHz, CDCl_3_) δ 4.95 (1H, dd, J = 4.5, 3.5 Hz), 4.55 (1H, s), 3.32 (4H, br s), 3.00 (1H, dd, J = 17.8, 3.5 Hz), 2.80 (1H, dd, J = 17.8, 4.5 Hz), 2.12−2.02 (2H, m), 1.99−1.90 (2H, m), 1.03 (21H, s); ^13^C-NMR (101 MHz, CDCl_3_) δ 173.9, 167.1, 100.6, 84.5, 83.3, 76.0 (CH-C5), 24.2, 18.7, 11.3; LRMS (CI, isobutane): *m*/*z* (int) 348 (98), 137 (61), 121 (51), 89 (94); HRMS (CI, isobutane) calculated for C_20_H_34_NO_2_Si [M + H]^+^: 348.2359; found 348.2364. Anal. calculated for C_20_H_33_NO_2_Si: C, 69.11; H, 9.57; N, 4.03. Found: C, 69.07; H, 9.65; N, 4.09.

### 4.2. Synthesis of 5-oxo-2-(3-triisopropylsilylprop-2-ynyl)-2,5-dihydrofuran-3-yl trifluoromethanesulfonate *(**3**)*

A solution of the lactone **2** (1.83 g, 5.27 mmol) was added to a solution of hydrochloric acid in EtOH (30 mL of a 1.2 m solution, 38 mmol, 7.1 equiv.) at 0 °C followed by water (3.0 mL). The mixture was heated at 78 °C for 5 h, cooled to room temperature and diluted with water (10 mL) and Et_2_O (40 mL). The phases were separated and the aqueous phase was extracted with Et_2_O (2 × 20 mL). The organic extracts were combined and washed with brine (20 mL), then dried over MgSO_4_, filtered and concentrated in vacuo. Azeotropic removal of water with toluene (3 × 50 mL) provided an orange residue that was used directly in the next step.

To a solution of the crude acid in CH_2_Cl_2_ (53 mL) at −78 °C was added dropwise freshly distilled Hünig’s base (1.40 mL, 8.03 mmol, 1.5 equiv.), and after 5 min triflic anhydride (4.15 mL, 6.96 mmol, 1.3 equiv.). The dark red solution was stirred at −78 °C for 1 h, then diluted with CH_2_Cl_2_ (30 mL) and warmed to room temperature. The reaction was quenched by the addition of water (20 mL) and the phases were separated. The aqueous phase was extracted with CH_2_Cl_2_ (2 × 20 mL), the organic extracts were combined, washed with brine (50 mL), dried with Na_2_SO_4_, filtered and concentrated in vacuo. The residue was purified by flash column chromatography (PE-Et_2_O, 9:1 → 4:1) to afford the triflate **3** (1.82 g, 81% over two steps) as a colourless solid. M.p. 42−43 °C; R*_f_* = 0.81 (PE-Et_2_O, 5:5); IR ν_max_ 2947, 2870, 2176, 1767, 1643, 995, 818 cm^−1^; ^1^H-NMR (400 MHz, CDCl_3_) δ 6.08 (1H, d, *J* = 1.2 Hz), 5.07 (1H, ddd, *J* = 4.8, 3.5, 1.2 Hz), 3.09 (1H, dd, *J* = 17.7, 4.8 Hz), 2.84 (1H, dd, *J* = 17.7, 3.5 Hz), 1.03 (21H, s); ^13^C-NMR (101 MHz, CDCl_3_) δ 167.8, 167.2, 118.6 (q, *J* = 322 Hz), 104.8, 97.3, 87.1, 76.3, 23.1, 18.6, 11.2; LRMS (CI, isobutane): *m*/*z* (*int*) 427 (26), 334 (11), 279 (32), 276 (21), 235 (15), 181 (11), 133 (27), 97 (35), 71 (100). HRMS (CI, isobutane) calculated for C_17_H_26_F_3_O_5_SSi [M + H]^+^: 427.1222; found 427.1223. Anal. calculated for C_17_H_25_F_3_O_5_SSi: C, 47.87; H, 5.91. Found: C, 47.85; H, 5.97.

### 4.3. Synthesis of 4-trimethylstannyl-5-(3-triisopropylsilylprop-2-yn-1-yl)-2,5-dihydrofuran-2-one (4)

In a 50 mL 3-necked flask containing thoroughly flame-dried LiCl (600 mg, 14.2 mmol, 8.0 equiv.) and Pd(PPh_3_)_4_ (80.1 mg, 0.0693 mmol, 3.9 mol %) was added THF (5 mL). A solution of triflate **3** (755 mg, 1.77 mmol) in THF (17 mL) was added. After 5 min hexamethylditin (480 µL, 2.31 mmol, 1.3 equiv.) was added and the mixture was heated at reflux for 1.5 h. Further Pd(PPh_3_)_4_ (67.8 mg, 0.0586 mmol, 3.3 mol %) was then added and the mixture was stirred for 1.5 h. The mixture was cooled to 0 °C and the reaction was quenched with saturated aqueous NaHCO_3_ (15 mL) and diluted with Et_2_O (40 mL). The phases were separated and the aqueous phase was extracted with Et_2_O (2 × 30 mL). The organic extracts were combined, washed with brine (40 mL), dried with Na_2_SO_4_, filtered and concentrated in vacuo. The crude product was purified by flash column chromatography (PE-Et_2_O, 19:1 → 4:1) to give the corresponding stannane **4** (419 mg, 54%) as a pale yellow solid. M.p. 69−72 °C; R_f_ = 0.21 (PE-Et_2_O, 4:1); IR ν_max_ 2940, 2863, 2176, 1751, 918, 880, 779 cm^−1^; ^1^H-NMR (400 MHz, CDCl_3_) δ 6.22 (1H, d, J = 1.9 Hz), 5.21 (1H, ddd, J = 5.3, 3.6, 1.9 Hz), 3.04 (1H, dd, J = 17.3, 5.3 Hz), 2.65 (1H, dd, J = 17.3, 3.6 Hz), 1.01 (21H, br s), 0.36 (9H, s); ^13^C-NMR (101 MHz, CDCl_3_) δ 175.4, 172.4, 131.1, 100.4, 85.8, 85.5, 25.1, 18.7, 11.3, −9.2; LRMS (CI, isobutane): m/z (int) 443 (76), 441 (57), 337 (11), 279 (48), 257 (15), 235 (12), 113 (20), 69 (100). HRMS (CI, isobutane) calculated for C_19_H_35_O_2_Si^120^Sn [M + H]^+^: 443.1429; found 443.1424.

### 4.4. Synthesis of 1-[4-(tert-butyldimethylsilyloxy)methylfuran-3-yl]butan-1-ol *(**6**)*

To a suspension of lithium aluminium hydride (4.74 g, 125 mmol, 2.3 equiv.) in THF (200 mL) at −78 °C was added a solution of dimethyl 3,4-furandicarboxylate (10.0 g, 54.3 mmol) in THF (200 mL) over 20 min at −78 °C. The solution was warmed gently to room temperature over 2 h and stirred overnight. The reaction was cooled to 0 °C and quenched carefully by success addition of water (4.7 mL), aqueous NaOH (1 m, 4.7 mL) and water (13 mL). After warming to room temperatureand stirring for 1 h, a cloudy white suspension was formed. MgSO_4_ (~15 g) was added and the mixture was filtered through a pad of Celite and washed with ethyl acetate (1 L). After concentration in vacuo, the pale-yellow oil obtained was used directly for the next step. 

To a solution of the crude diol obtained (6.95 g, ~54.2 mmol) in CH_2_Cl_2_ (450 mL) was added activated manganese(II) oxide (28.3 g, 326 mmol, 6.0 equiv.) at room temperature. The mixture was stirred vigorously for 2.5 h and further manganese(II) oxide (9.50 g, 108 mmol, 2.0 equiv.) was added three times at regular intervals. The black suspension was then filtered through a pad of Celite and washed with CH_2_Cl_2_ (1.5 L). After concentration in vacuo, the crude yellow oil was separated into two fractions—A (3.46 g) and B (3.72 g)—which were used in the subsequent steps without any further purification.

To a solution of the crude aldehyde A (3.46 g, ~27.5 mmol) in CH_2_Cl_2_ (250 mL), imidazole (2.24 g, 33.0 mmol, 1.2 equiv.), DMAP (336 mg, 2.75 mmol, 0.10 equiv.) and t-butyldimethylsilyl chloride (4.55 g, 30.2 mmol, 1.1 equiv.) were added sequentially. The solution was stirred for 20 min at room temperature and then water (60 mL) was added. The phases were separated and the aqueous phase was extracted with CH_2_Cl_2_ (2 × 60 mL). The organic extracts were combined, washed with brine (100 mL), dried over MgSO_4_, filtered and concentrated in vacuo. The resulting pale yellow oil was used immediately in the next step.

To a solution of the silylated aldehyde **5** (~27.5 mmol) in THF (250 mL) at −78 °C was added dropwise n-propylmagnesium chloride (23.3 mL of a 2.0 m solution in THF, 46.6 mmol, 1.7 equiv.). The solution was stirred at −78 °C for 1.5 h, warmed to 0 °C and the reaction was quenched by the addition of a saturated aqueous solution of NH_4_Cl (35 mL). Water (35 mL) and Et_2_O (60 mL) were added and the phases separated. The aqueous phase was extracted with Et_2_O (2 × 60 mL) and the organic extracts washed with brine (120 mL), dried over MgSO_4_, filtered and concentrated in vacuo. The residual material was purified by flash column chromatography (PE-Et_2_O, 9:1) to give the desired alcohol **6** as a colourless oil.

The same procedure was used to convert aldehyde B in the alcohol **6** and the batches were combined (10.7 g, 69% over 4 steps). R*_f_* = 0.34 (PE-Et_2_O, 9:1); IR ν_max_ 3349, 2955, 2929, 2858, 1749, 1669, 960, 877, 815, 777, 760, 742 cm^−1^; ^1^H-NMR (400 MHz, CDCl_3_) δ 7.30 (2H, s), 4.65 (1H, d, *J* = 12.2 Hz), 4.61 (1H, d, *J* = 12.2 Hz), 4.60 (1H, dt, *J* = 7.9, 5.6 Hz), 3.58 (1H, d, *J* = 5.6 Hz), 1.84 (1H, dddd, *J* = 13.4, 9.8, 7.9, 5.5 Hz), 1.73 (1H, dddd, *J* = 13.4, 9.8, 5.9, 5.6 Hz), 1.57−1.48 (1H, m), 1.43−1.34 (1H, m), 0.96 (3H, t, *J* = 7.4 Hz), 0.91 (9H, s), 0.12 (3H, s), 0.12 (3H, s); ^13^C-NMR (101 MHz, CDCl_3_) δ 140.6, 140.1, 128.5, 123.7, 65.8, 56.8, 38.5, 26.0, 19.5, 18.4, 14.1, −5.2; LRMS (CI, isobutane): *m/z* (*int*) 267 (31), 135 (49), 107 (9), 89 (100), 69 (20). HRMS (CI, isobutane) calculated for C_15_H_27_O_2_Si [M − OH]^+^: 267.1780; found 267.1775.

### 4.5. Synthesis of 1-[4-(tert-butyldimethylsilyloxy)methylfuran-3-yl]butan-1-one *(**7**)*

To a solution of alcohol **6** (522 mg, 1.94 mmol) in CH_2_Cl_2_ (20 mL) was added activated manganese(II) oxide (3.43 g, 39.5 mmol, 20 equiv.). The mixture was stirred at room temperature for 2 h, then heated at reflux for 2 h, and stirred overnight at room temperature. The suspension was filtered through a pad of Celite and washed with CH_2_Cl_2_ (500 mL). The filtrate was concentrated in vacuo and the residue was purified by flash column chromatography (PE-Et_2_O, 19:1), affording the desired ketone **7** (468 mg, 90%) as a colourless oil. R*_f_* = 0.63 (PE-Et_2_O, 9:1); IR ν_max_ 2957, 2930, 2886, 2859, 2361, 1676, 837, 814, 777 cm^−1^; ^1^H-NMR (400 MHz, CDCl_3_) δ 7.98 (1H, d, *J* = 1.7 Hz), 7.40 (1H, q, *J* = 1.7 Hz), 4.87 (2H, d, *J* = 1.7 Hz), 2.69 (2H, t, *J* = 7.3 Hz), 1.72 (2H, qt, *J* = 7.4, 7.3 Hz), 0.97 (3H, t, *J* = 7.4 Hz), 0.93 (9H, s), 0.10 (6H, s); ^13^C-NMR (101 MHz, CDCl_3_) δ 196.3, 148.5, 141.6, 127.1, 125.4, 58.8, 42.3, 26.1, 18.5, 17.9, 14.0, −5.3; LRMS (CI, isobutane): *m/z* (*int*) 283 (46), 225 (8), 89 (100), 69 (10). HRMS (CI, isobutane) calculated for C_15_H_27_O_3_Si [M + H]^+^: 283.1729; found 283.1732.

### 4.6. Synthesis of tert-butyldimethyl{[4-(pent-1-en-2-yl)furan-3-yl]methoxy}silane *(**8**)*

To a suspension of methyltriphenylphosphonium bromide (2.87 g, 8.03 mmol, 5.0 equiv.) in THF (7.5 mL) at 0 °C was added dropwise a solution of NaHMDS (6.4 mL of a 1.0 m solution in THF, 6.4 mmol, 4.0 equiv.). The bright yellow suspension was warmed to room temperature and stirred for 1 h, before being cooled to 0 °C. A solution of ketone **7** (453 mg, 1.60 mmol) in THF (7 mL) was added dropwise and the mixture was stirred for 1 h at room temperature. The reaction was quenched by the addition of a saturated aqueous solution of NH_4_Cl (10 mL) and the mixture was diluted with Et_2_O (30 mL). The phases were separated and the aqueous phase was extracted with Et_2_O (2 × 20 mL). The organic extracts were combined, washed with brine (40 mL), dried over MgSO_4_, filtered and concentrated in vacuo. The residue was purified by flash column chromatography (PE-Et_2_O, 99:1) to give the corresponding 1,1-disubstituted alkene **8** (426 mg, 95%) as a colourless oil. R*_f_* = 0.91 (PE-Et_2_O, 19:1); IR ν_max_ 2957, 2930, 2857, 1636, 874, 833, 814, 793, 773, 736 cm^−1^; ^1^H-NMR (400 MHz, CDCl_3_) δ 7.36 (1H, s), 7.34 (1H, s), 5.12 (1H, s), 5.00 (1H, d, *J* = 0.9 Hz), 4.63 (2H, s), 2.29 (2H, t, *J* = 7.4 Hz), 1.51 (2H, qt, *J* = 7.4, 7.4 Hz), 0.92 (3H, t, *J* = 7.4 Hz), 0.91 (9H, s), 0.08 (6H, s); ^13^C-NMR (101 MHz, CDCl_3_) δ 141.4, 140.5, 139.8, 125.7, 124.7, 112.6, 57.6, 38.6, 26.0, 21.5, 18.5, 14.0, −5.1; HRMS (ESI) calculated for C_16_H_28_NaO_2_Si [M + Na]^+^: 303.1751; found 303.1744.

### 4.7. Synthesis of 2-[4-(tert-butyldimethylsilyloxymethyl)furan-3-yl]pentan-1-ol *(**9**)*

To a solution of 1,1-disubstituted alkene **8** (1.07 g, 3.81 mmol) in THF (4 mL), cooled to 0 °C, was added dropwise a solution of 9-BBN (23.0 mL of a 0.5 m solution in THF, 11.5 mmol, 3.0 equiv.). The mixture was warmed to 65 °C for 75 min and then cooled to 0 °C before careful addition of EtOH (18 mL) and aqueous 3 m NaOH (11.5 mL). After 15 min, aqueous hydrogen peroxide (30%, 18 mL) was added. The mixture was heated at reflux for 1 h, cooled to room temperature before the addition of Et_2_O (60 mL) and water (20 mL). The phases were separated and the aqueous phase was extracted with Et_2_O (2 × 50 mL). The organic extracts were combined and washed with brine, dried over MgSO_4_, filtered and concentrated in vacuo. The crude product was purified by flash column chromatography (PE-Et_2_O, 9:1) to give the desired primary alcohol **9** (1.01 g, 89%) as a colourless oil. R*_f_* = 0.18 (PE-Et_2_O, 9:1); IR ν_max_ 3381, 2955, 2930, 2895, 2858, 1602, 1541, 871, 837, 775 cm^−1^; ^1^H-NMR (400 MHz, CDCl_3_) δ 7.33 (1H, d, *J* = 1.6 Hz), 7.23 (1H, d, *J* = 1.6 Hz), 4.56 (1H, d, *J* = 12.5 Hz), 4.53 (1H, d, *J* = 12.5 Hz), 3.73 (1H, ddd, *J* = 10.8, 6.4, 4.9 Hz), 3.61 (1H, ddd, *J* = 10.8, 7.3, 6.0 Hz), 2.83−2.75 (1H, m), 2.17 (1H, dd, *J* = 6.4, 6.0 Hz), 1.67−1.57 (1H, m), 1.56−1.48 (1H, m), 1.38−1.27 (2H, m), 0.91 (9H, s), 0.89 (3H, t, *J* = 5.9 Hz), 0.10 (6H, s); ^13^C-NMR (126 MHz, CDCl_3_) δ 141.1, 140.6, 125.6, 124.9, 66.8, 56.5, 38.1, 33.8, 26.0, 20.8, 18.5, 14.2, −5.2; HRMS (ESI) calculated for C_16_H_30_NaO_3_Si [M + Na]^+^: 321.1856; found 321.1846.

### 4.8. Synthesis tert-butyl{[4-(1-tert-butyldiphenylsilyloxypentan-2-yl)furan-3-yl]methoxy}dimethylsilane *(**10**)*

To a solution of alcohol **9** (1.72 g, 5.76 mmol) in CH_2_Cl_2_ (58 mL), DMAP (203 mg, 1.66 mmol, 0.3 equiv.), *t*-butyldiphenylsilyl chloride (2.26 mL, 8.81 mmol, 1.5 equiv.) and Et_3_N (1.37 mL, 9.83 mmol, 1.7 equiv.) were added successively at 0 °C. The solution was stirred overnight at room temperature and the reaction was quenched with a saturated aqueous solution of NH_4_Cl (20 mL). The phases were separated and the aqueous phase was extracted with CH_2_Cl_2_ (2 × 30 mL). The organic extracts were combined and then washed with brine (40 mL), dried over MgSO_4_, filtered and concentrated in vacuo. The crude product was purified by flash column chromatography (PE-CH_2_Cl_2_, 9:1) to afford furan **10** (2.81 g, 91%) as a colourless oil. R*_f_* = 0.50 (PE-CH_2_Cl_2_, 8:2); IR ν_max_ 3073, 3050, 2955, 2930, 2857, 1589, 1541, 835, 775, 739, 700 cm^−1^; ^1^H-NMR (400 MHz, CDCl_3_) δ 7.61−5.56 (4H, m), 7.44−7.33 (6H, m), 7.27 (1H, d, *J* = 0.8 Hz), 7.17 (1H, d, *J* = 0.8 Hz), 4.45 (2H, s), 3.67 (1H, dd, *J* = 10.1, 5.8 Hz), 3.63 (1H, dd, *J* = 10.1, 6.4 Hz), 2.75 (1H, dddd, *J* = 8.6, 6.4, 6.1, 5.8 Hz), 1.78 (1H, app ddt, *J* = 13.3, 9.7, 6.1 Hz), 1.54−1.44 (1H, m), 1.35−1.20 (2H, m), 1.02 (9H, s), 0.90−0.84 (12H, m), 0.04 (3H, s), 0.03 (3H, s); ^13^C-NMR (101 MHz, CDCl_3_) δ 140.4, 139.9, 135.8, 135.7, 133.9, 129.7, 129.7, 127.7, 125.6, 125.5, 67.3, 56.9, 37.5, 34.0, 27.0, 26.0, 20.5, 19.4, 18.4, 14.4, −5.2; LRMS (CI, isobutane): *m*/*z* (*int*) 537 (5), 461 (27), 447 (12), 405 (100), 133 (13). HRMS (CI, isobutane) calculated for C_32_H_49_O_3_Si_2_ [M + H]^+^: 537.3220; found 537.3221.

### 4.9. Synthesis of [4-(1-tert-butyldiphenylsilyloxypentan-2-yl)furan-3-yl]methanol *(**11**)*

To a solution of furan **10** (2.81 g, 5.23 mmol) in ethanol (18 mL) was added PPTS (660 mg, 2.63 mmol, 0.5 equiv.) and the mixture was stirred overnight at 40 °C. The reaction was quenched with a saturated aqueous solution of NaHCO_3_ (10 mL) and the ethanol was removed in vacuo. The residue was diluted with Et_2_O (20 mL) and the phases were separated. The aqueous phase was extracted with Et_2_O (2 × 10 mL) and the combined organic extracts were washed with brine (20 mL), dried over MgSO_4_, filtered and concentrated in vacuo. Residual material was purified by flash column chromatography (PE-Et_2_O, 9:1) to give the desired alcohol **11** (2.09 g, 94%) as a colourless oil. R*_f_* = 0.20 (PE-Et_2_O, 9:1); IR ν_max_ 3366, 2042, 2893, 2866, 1757, 1600, 1541 cm^−1^; ^1^H-NMR (400 MHz, CDCl_3_) δ 7.62−7.53 (4H, m), 7.45−7.34 (7H, m), 7.19 (1H, d, *J* = 1.5 Hz), 4.42 (2H, d, *J* = 5.4 Hz), 3.72 (1H, dd, *J* = 9.8, 5.7 Hz), 3.63 (1H, dd, *J* = 9.8, 6.9 Hz), 2.81 (1H, ddt, *J* = 9.1, 6.9, 5.7 Hz), 1.95 (1H, t, *J* = 5.4 Hz), 1.69 (1H, dddd, *J* = 13.1, 9.5, 6.4, 5.7 Hz), 1.54−1.42 (1H, m), 1.37−1.20 (2H, m), 1.02 (9H, s), 0.87 (3H, t, *J* = 7.3 Hz); ^13^C-NMR (101 MHz, CDCl_3_) δ 140.5, 140.5, 135.8, 135.7, 133.4, 133.4, 129.8, 127.8, 127.7, 126.1, 125.4, 68.7, 55.5, 37.3, 34.0, 26.9, 20.6, 19.3, 14.3; LRMS (CI, isobutane): *m*/*z* (*int*) 405 (34), 365 (6), 341 (5), 265 (100), 237 (12), 217 (75) 135 (9). HRMS (CI, isobutane) calculated for C_26_H_33_O_2_Si [M − OH]^+^: 405.2250; found 405.2252.

### 4.10. Synthesis of tert-butyl[2-(4-chloromethylfuran-3-yl)pentyloxy]diphenylsilane *(**12**)*

To a solution of the alcohol **11** (1.47 g, 3.48 mmol) in CH_2_Cl_2_ (12 mL) cooled to 0 °C, Et_3_N (870 μL, 6.24 mmol, 1.8 equiv.) and MsCl (405 μL, 5.23 mmol, 1.5 equiv.), both freshly distilled, were added successively. The mixture was warmed to room temperature, stirred overnight and the reaction was quenched by the addition of a saturated aqueous solution of NH_4_Cl (10 mL). The phases were separated and the aqueous phase was extracted with CH_2_Cl_2_ (2 × 15 mL). The organic extracts were combined and dried over MgSO_4_, filtered and concentrated in vacuo. The crude product was purified by flash column chromatography (PE-CH_2_Cl_2_, 9:1) to deliver the chloride **12** (1.38 g, 90%) as a colourless oil. R*_f_* = 0.40 (PE-CH_2_Cl_2_, 9:1); IR ν_max_ 3071, 2943, 2910, 2862, 1589, 1543, 864, 702 cm^−1^; ^1^H-NMR (400 MHz, CDCl_3_) δ 7.61−7.57 (4H, m), 7.44−7.33 (7H, m), 7.20 (1H, d, *J* = 1.5 Hz), 4.35 (2H, s), 3.67 (2H, d, *J* = 5.8 Hz), 2.82 (1H, dtd, *J* = 9.0, 5.8, 5.7 Hz), 1.81 (1H, dddd, *J* = 13.2, 9.3, 6.5, 5.7 Hz), 1.59−1.46 (1H, m), 1.37−1.25 (2H, m), 1.03 (9H, s), 0.90 (3H, t, *J* = 7.3 Hz); ^13^C-NMR (101 MHz, CDCl_3_) δ 141.5, 140.9, 135.8, 135.7, 133.8, 133.7, 129.8, 129.7, 127.8, 126.1, 122.5, 67.8, 37.2, 36.4, 34.1, 27.0, 20.6, 19.4, 14.3; LRMS (CI, isobutane): *m*/*z* (*int*) 441 (60), 405 (98), 363 (72), 241 (61), 227 (77), 185 (100), 149 (45), 91 (31). HRMS (CI, isobutane) calculated for C_26_H_34_O_2_^35^ClSi [M + H]^+^: 441.2017; found 441.2015. Anal. calculated for C_26_H_33_O_2_ClSi: C, 70.80; H, 7.54. Found: C, 70.93; H, 7.59.

### 4.11. Synthesis of 4-[4-(1-tert-butyldiphenylsilyloxypentan-2-yl)furan-3-yl]methyl-5-[3-triisopropylsilyl-prop-2-yn-1-yl]-2,5-dihydrofuran-2-one *(**13**)*

To a solution of chloride **12** (449 mg, 1.02 mmol) in THF (2 mL) was added Pd_2_(dba)_3_ (57 mg, 0.062 mmol, 6.6 mol %) and triphenylarsine (106 mg, 0.346 mmol, 0.36 equiv.). The purple to yellow mixture was stirred for 5 min at room temperature before a solution of the stannane **4** (419 mg, 0.950 mmol) in THF (9 mL) was added. The mixture was heated at 65 °C overnight, cooled to room temperature and then diluted with Et_2_O (30 mL) and H_2_O (10 mL). The phases were separated and the aqueous phase was extracted with Et_2_O (2 × 20 mL). The organic extracts were combined, washed with brine (30 mL), dried over MgSO_4_, filtered and concentrated in vacuo. Residual material was purified by flash column chromatography (PE−Et_2_O, 95:5→92:8) to give the corresponding product **13** (605 mg, 93%) as a pale-yellow oil and an inseparable mixture (1:1) of diastereoisomers. R*_f_* = 0.69 (PE−Et_2_O, 7:3); IR ν_max_ 3071, 2932, 2862, 2175, 1759, 1643, 1589, 995, 926, 872, 802, 741, 702, 679 cm^−1^; ^1^H-NMR (400 MHz, CDCl_3_) δ 7.58−7.54 (8H, m), 7.45−7.33 (12H, m), 7.25 (1H, d, *J* = 1.6 Hz), 7.24−7.23 (2H, m), 7.22 (1H, d, *J* = 1.5 Hz), 5.72 (1H, d, *J* = 1.3 Hz), 5.67 (1H, d, *J* = 1.5 Hz), 4.82 (1H, app t, *J* = 5.0 Hz), 4.80 (1H, app t, *J* = 5.0 Hz), 3.62−3.59 (4H, m), 3.52 (1H, d, *J* = 17.7 Hz), 3.43 (1H, d, *J* = 18.4 Hz), 3.24 (1H, dd, *J* = 17.7, 1.5 Hz), 3.20 (1H, d, *J* = 18.4 Hz), 2.90 (1H, dd, *J* = 5.1, 2.0 Hz), 2.86 (1H, dd, *J* = 5.1, 2.0 Hz), 2.67 (1H, dd, *J* = 5.1, 4.0 Hz), 2.63 (1H, dd, *J* = 5.1, 3.9 Hz), 2.54 (1H, dq, *J* = 9.0, 5.8 Hz), 2.49 (1H, dq, *J* = 8.7, 5.8 Hz), 1.79−1.69 (2H, m), 1.51−1.38 (2H, m), 1.30−1.16 (4H, m), 1.05−0.98 (60H, m), 0.86 (3H, t, *J* = 7.3 Hz), 0.87 (3H, t, *J* = 7.3 Hz); ^13^C-NMR (101 MHz, CDCl_3_) δ 171.9, 169.7, 169.5, 140.8, 140.7, 140.4, 140.3, 135.7, 135.6, 133.6, 133.5, 129.9, 129.8, 127.8, 126.2, 119.4, 118.4, 99.7, 99.6, 85.4, 80.5, 80.4, 67.9, 67.8, 37.5, 34.2, 34.1, 27.0, 23.6, 23.5, 23.1, 23.0, 20.7, 19.4, 18.7, 14.4, 11.3; LRMS (FAB): *m*/*z* (*int*) 705 (100), 605 (67), 427 (9), 197 (40), 135 (73), 59 (42). HRMS (FAB) calculated for C_42_H_58_NaO_4_Si_2_ [M+Na]^+^: 705.3771; found 705.3763.

### 4.12. Synthesis of 4-[4-(1-hydroxypentan-2-yl)furan-3-yl]methyl-5-(prop-2-yn-1-yl)-2,5-dihydrofuran-2-one *(**14**)*

To a solution of **13** (1.23 g, 1.80 mmol) in THF (31 mL) at 0 °C was added acetic acid (400 µL, 6.99 mmol, 3.88 equiv.) and TBAF (7.4 mL of a 1 m solution in THF, 7.4 mmol, 4.1 equiv.). The mixture was warmed slowly to room temperature, stirred for 24 h and then diluted with water (10 mL) and ethyl acetate (30 mL). The phases were separated and the aqueous phase was extracted with ethyl acetate (2 × 30 mL). The organic extracts were combined, washed with brine (50 mL), dried over MgSO_4_, filtered and concentrated in vacuo. Residual material was purified by flash column chromatography (PE−Et_2_O, 1:1→1:4) to give a diastereomeric mixture of the corresponding unprotected alcohols **14** (461 mg, 89%) as a pale-yellow oil and a partially separable 1:1 mixture of diastereoisomers. R*_f_* = 0.44 and 0.42 (PE-Et_2_O, 1:4); IR ν_max_ 3446, 2954, 2933, 2869, 2360, 1742, 1645, 924, 875, 850, 798 cm^−1^; Less polar diastereoisomer ^1^H-NMR (400 MHz, CDCl_3_) δ 7.30 (2H, s), 5.78 (1H, ddd, *J* = 1.5, 1.5, 1.2 Hz), 5.03 (1H, ddd, *J* = 5.6, 4.2, 1.2 Hz), 3.68−3.60 (2H, m), 3.59 (1H, dd, *J* = 18.3, 1.5 Hz), 3.45 (1H, dd, J = 18.3, 1.5 Hz), 2.85 (1H, ddd, *J* = 17.3, 5.6, 2.6 Hz), 2.73 (1H, ddd, *J* = 17.3, 4.2, 2.7 Hz), 2.59 (1H, dq, *J* = 8.6, 5.9 Hz), 2.07 (1H, dd, *J* = 2.7, 2.6 Hz), 1.69−1.59 (1H, m), 1.52−1.41 (1H, m), 1.36−1.23 (2H, m), 0.89 (3H, t, *J* = 7.3 Hz); Less polar diastereoisomer ^13^C-NMR (101 MHz, CDCl_3_) δ 172.0, 169.9, 141.0, 140.5, 126.0, 119.5, 118.5, 80.3, 76.6, 72.6, 66.7, 37.5, 34.5, 23.2, 22.6, 20.7, 14.3; More polar diastereoisomer ^1^H-NMR (400 MHz, CDCl_3_) δ 7.30 (2H, s), 5.81 (1H, ddd, *J* = 1.6, 1.6, 1.5 Hz), 5.00 (1H, ddd, *J* = 6.3, 4.6, 1.5 Hz), 3.70−3.59 (2H, m), 3.59 (1H, dd, *J* = 18.2, 1.6 Hz), 3.44 (1H, dd, *J* = 18.2, 1.6 Hz), 2.87 (1H, ddd, *J* = 17.3, 6.3, 2.5 Hz), 2.74 (1H, ddd, *J* = 17.3, 4.6, 2.5 Hz), 2.68−2.55 (1H, m), 2.08 (1H, t, *J* = 2.5 Hz), 1.69−1.58 (1H, m), 1.52−1.40 (1H, m), 1.36−1.27 (2H, m), 0.89 (3H, t, *J* = 7.3 Hz,); More polar diastereoisomer ^13^C-NMR (101 MHz, CDCl_3_) δ 172.0, 169.7, 141.1, 140.6, 126.0, 119.4, 118.5, 80.2, 76.5, 72.7, 66.9, 37.5, 34.2, 23.2, 22.6, 20.6, 14.3; LRMS (CI, isobutane): *m*/*z* (int) 289 (100), 71 (13). HRMS (CI, isobutane) calculated for C_17_H_21_O_4_ [M + H]^+^: 289.1440; found 289.1442.

### 4.13. Synthesis of (5S*)-4-{4-[(2S*)-1-Hydroxypentan-2-yl]furan-3-yl}methyl-5-(2-iodoprop-2-en-1-yl)-2,5-dihydrofuran-2-one *(**15a**)* and (5R*)-4-{4-[(2S*)-1-Hydroxypentan-2-yl]furan-3-yl}methyl-5-(2-iodoprop-2-en-1-yl)-2,5-dihydrofuran-2-one *(**15b**)*

To a solution of alkynes **14** (419 mg, 1.45 mmol) in THF (6.6 mL) at room temperature was added Pd(PPh_3_)_4_ (60.1 mg, 0.0520 mmol, 3.6 mol %) followed by tributyltin hydride (425 µL, 1.58 mmol, 1.1 equiv.). The mixture was stirred for 20 min and the reaction was quenched with saturated aqueous NaHCO_3_ (5 mL). The mixture was diluted with Et_2_O (10 mL) and the phases were then separated. The aqueous phase was extracted with Et_2_O (3 × 10 mL) and the organic extracts were combined, washed with brine (20 mL), dried with Na_2_SO_4_, filtered and concentrated in vacuo. The crude material was purified by flash column chromatography (PE-Et_2_O, 1:1→2:3) to give a partially separable regioisomeric mixture (3.2:1 ratio, estimated by ^1^H-NMR analysis) in favour of the required vinylic stannane. Most of the required stannane (572 mg) were isolated and taken straight to the next step for better characterisation. R_f_ = 0.68 and 0.76 (PE-Et_2_O, 1:4).

To a solution of the vinylic stannane (572 mg, 0.988 mmol) in CH_2_Cl_2_ (10 mL) at 0 °C was added I_2_ (289 mg, 1.14 mmol, 1.15 equiv.). The mixture was stirred for 20 min and the reaction was then quenched with a saturated aqueous solution of Na_2_S_2_O_3_ (15 mL). The mixture was diluted with CH_2_Cl_2_ (20 mL) and after 10 min, the phases were separated. The aqueous phase was extracted with CH_2_Cl_2_ (2 × 20 mL) and the organic extracts were combined, washed with brine (30 mL), dried with Na_2_SO_4_, filtered and concentrated in vacuo. The crude product was purified by flash column chromatography (PE-Et_2_O, 55:45) to give the separable diastereomeric vinyl iodides **15a** and **15b** (378 mg, 62% combined over two steps) as colourless oils. **15a** (*less polar diastereomer*) R*_f_* = 0.36 (PE-Et_2_O, 1:4); IR ν_max_ 3446, 2955, 2929, 2870, 1743, 1637, 1618, 1541, 960, 912, 871, 857, 840, 799 cm^−1^; ^1^H-NMR (400 MHz, CDCl_3_) δ 7.30 (2H, s), 6.25 (1H, d, *J* = 1.7 Hz), 5.91 (1H, d, *J* = 1.7 Hz), 5.75 (1H, ddd, *J* = 1.4, 1.1, 1.1 Hz), 5.16 (1H, ddd, *J* = 8.7, 3.6, 1.4 Hz), 3.65 (1H, dd, *J* = 10.5, 5.6 Hz), 3.59 (1H, dd, *J* = 10.5, 6.8 Hz), 3.55 (1H, dd, *J* = 17.8, 1.1 Hz), 3.46 (1H, dd, *J* = 17.8, 1.1 Hz), 2.94 (1H, dd, *J* = 15.0, 3.6 Hz), 2.64 (1H, dd, *J* = 15.0, 8.7 Hz), 2.56 (1H, dddd, *J* = 8.5, 6.8, 5.9, 5.6 Hz), 1.69−1.58 (2H, m), 1.51−1.40 (1H, m), 1.36−1.23 (2H, m), 0.89 (3H, t, *J* = 7.3 Hz); ^13^C-NMR (101 MHz, CDCl_3_) δ 172.0, 170.2, 141.0, 140.5, 130.5, 126.0, 119.4, 118.3, 102.1, 81.9, 66.8, 48.2, 37.6, 34.4, 23.4, 20.7, 14.3; LRMS (EI+): *m*/*z* (*int*) 416 (100), 385 (10), 289 (56), 271 (20), 215 (33), 161 (35), 119 (21), 91 (45), 77 (20), 55 (10). HRMS (EI+) calculated for C_17_H_21_IO_4_ [M]^+^: 416.0485; found 416.0488. **15b** (*more polar diastereomer*) R*_f_* = 0.31 (PE-Et_2_O, 2:8); IR ν_max_ 3446, 2955, 2928, 2869, 1746, 1638, 1618, 1539, 914, 870, 857, 840, 799 cm^−1^; ^1^H-NMR (400 MHz, CDCl_3_) δ 7.30 (2H, s), 6.26 (1H, dd, *J* = 1.7, 0.8 Hz), 5.91 (1H, d, *J* = 1.7 Hz), 5.78 (1H, ddd, *J* = 1.5, 1.4, 1.1 Hz), 5.13 (1H, ddd, *J* = 8.6, 3.6, 1.5 Hz), 3.67 (1H, dd, *J* = 10.5, 5.5 Hz), 3.60 (1H, dd, *J* = 18.1, 1.1 Hz), 3.59 (1H, dd, *J* = 10.5, 6.9 Hz), 3.44 (1H, dd, *J* = 18.1, 1.4 Hz), 2.95 (1H, dd, *J* = 15.0, 3.6 Hz), 2.63 (1H, ddd, *J* = 15.0, 8.6, 0.8 Hz), 2.57 (1H, dddd, *J* = 8.6, 6.9, 5.6, 5.5 Hz), 1.63 (1H, dddd, *J* = 13.1, 9.6, 6.1, 5.6 Hz), 1.55 (1H, br s), 1.51−1.41 (1H, m), 1.36−1.22 (2H, m), 0.89 (3H, t, *J* = 7.3 Hz); ^13^C-NMR (101 MHz, CDCl_3_) δ 172.0, 170.1, 141.0, 140.6, 130.5, 126.0, 119.4, 118.3, 102.2, 81.8, 67.0, 48.2, 37.6, 34.2, 23.4, 20.7, 14.3; LRMS (CI, isobutane): *m*/*z* (*int*) 417 (69), 291 (100), 273 (46), 251 (10), 97 (13), 71 (28). HRMS (CI, isobutane) calculated for C_17_H_22_IO_4_ [M + H]^+^: 417.0563; found 417.0564.

### 4.14. Synthesis of (8S*,9R*,12S*)-9-Hydroxy-10-methylidene-8-propyl-5,13-dioxatricyclo[10.3.0.03,7]penta-deca-1(15),3,6-trien-14-one *(**16a**)* and (8S*,9S*,12S*)-9-Hydroxy-10-methylidene-8-propyl-5,13-dioxa-tricyclo[10.3.0.03,7]pentadeca-1(15),3,6-trien-14-one *(**16b**)*

To a solution of the alcohol **15a** (59.2 mg, 0.142 mmol) in CH_2_Cl_2_ (2.8 mL) was added Dess-Martin periodinane (132 mg, 0.311 mmol, 2.2 equiv.). The mixture was stirred at room temperature for 2 h and then cooled to 0 °C. The reaction was quenched by the addition of a saturated aqueous solution of Na_2_S_2_O_3_ (5 mL) and the mixture was diluted with water (5 mL) and CH_2_Cl_2_ (5 mL). After 10 min, the phases were separated and the aqueous phase was extracted with CH_2_Cl_2_ (3 × 5 mL). The organic extracts were combined and washed with brine (10 mL), then dried with Na_2_SO_4_, filtered and concentrated in vacuo. The crude product was purified quickly by passage through a small plug of silica (PE-Et_2_O, 4:1) to afford the corresponding aldehyde (50.1 mg, 85%) as a colourless oil.

To a solution of CrCl_2_ (200 mg, 1.63 mmol, 13.5 equiv.) and NiCl_2_ (1.6 mg, 0.012 mmol, 10 mol %) in previously degassed (three freeze-thaw cycles) DMSO (6 mL) was added a solution of aldehyde (50.1 mg) in degassed (three freeze-thaw cycles) DMSO (6 mL) at room temperature. The dark green mixture was stirred at room temperature for 24 h and at 50 °C for 16 h. The reaction was quenched by the addition of a saturated aqueous solution of NH_4_Cl (15 mL) and the mixture was diluted with EtOAc (30 mL). The biphasic mixture was stirred for 30 min, the phases were separated and the aqueous phase was extracted with EtOAc (3 × 30 mL). The organic extracts were combined, washed with brine (50 mL), dried over MgSO_4_, filtered and concentrated in vacuo. Residual material was purified by flash column chromatography (PE-Et_2_O, 9:11) allowing the separation of minor diastereomer **16b** (5.5 mg, 13% over two steps) as a colourless oil and major diastereomer **16a** (20.2 mg, 49% over two steps) as a colourless solid. The crystal structure of the major alcohol product **16a** was obtained. **16b** (less polar, minor diastereomer) M.p. 140−143 °C; R*_f_* = 0.53 (PE-Et_2_O, 1:4); IR ν_max_ 3454, 2955, 2930, 2870, 1743, 1636, 1537, 926, 868, 802, 766, 739 cm^−1^; ^1^H-NMR (500 MHz, CDCl_3_) δ 7.37 (1H, d, *J* = 1.5 Hz), 7.23 (1H, d, *J* = 1.5 Hz), 5.89 (1H, ddd, *J* = 1.6, 1.1, 1.1 Hz), 5.23 (1H, ddd, *J* = 3.6, 2.7, 1.6 Hz), 4.97 (1H, s), 4.94 (1H, s), 4.12 (1H, d, *J* = 1.5 Hz), 3.68 (1H, dd, *J* = 14.7, 1.1 Hz), 3.01 (1H, dd, *J* = 14.7, 1.1 Hz), 2.97 (1H, ddd, *J* = 8.6, 6.5, 1.5 Hz), 2.91 (1H, dd, *J* = 16.4, 2.7 Hz), 2.45 (1H, dd, *J* = 16.4, 3.6 Hz), 1.84−1.65 (2H, m, CH_2_-C7), 1.57 (1H, br s), 1.40−1.29 (2H, m), 0.92 (3H, t, *J* = 7.4 Hz); ^13^C-NMR (126 MHz, CDCl_3_) δ 172.6, 170.3, 143.2, 140.8, 140.5, 122.5, 122.0, 120.0, 113.5, 83.9, 74.8, 38.0, 30.5, 30.2, 21.5, 21.1, 14.1; LRMS (CI, isobutane): *m*/*z* (*int*) 289 (72), 273 (12), 137 (13), 113 (68), 97 (68), 81 (73), 71 (100). HRMS (CI, isobutane) calculated for C_17_H_21_O_4_ [M+H]^+^: 289.1440; found 289.1438. **16a** (*more polar, major diastereomer*) M.p. 140−142 °C; R*_f_* = 0.44 (PE-Et_2_O, 2:8); IR ν_max_ 3400, 2957, 2928, 2872, 1736, 1636, 1537, 962, 939, 910, 870, 802, 777, 739 cm^−1^; ^1^H-NMR (500 MHz, CDCl_3_) δ 7.34 (1H, s), 7.23 (1H, s), 5.93 (1H, s), 5.15 (1H, s), 4.92 (1H, s), 4.87 (1H, s), 3.90 (1H, d, *J* = 4.3 Hz), 3.68 (1H, d, *J* = 16.0 Hz), 3.27 (1H, br s), 2.81−2.71 (2H, m), 2.55 (1H, br s), 1.85 (2H, br s), 1.55−1.44 (1H, m), 1.39−1.28 (1H, m), 1.27−1.14 (1H, m), 0.89 (3H, t, *J* = 7.4 Hz); ^13^C-NMR (126 MHz, CDCl_3_) δ 172.8, 170.4, 141.9, 141.5, 141.0, 123.3, 121.1, 119.7, 118.6, 83.2, 81.5, 39.7, 32.7, 28.9, 22.8, 20.9, 14.2; LRMS (EI+): *m*/*z* (*int*) 288 (100), 270 (43), 259 (36), 219 (64), 173 (51), 129 (39), 91 (81), 77 (47), 43 (47). HRMS (EI+) calculated for C_17_H_20_O_4_ [M]^+^: 288.1362; found 288.1360. X-ray crystal data (CCDC 1920025) for C_17_H_20_O_4_ (*M* = 288.34 g/mol): orthorhombic, space group P212121, a = 7.2695(5) Å, b = 11.8269(10) Å, c = 17.3278(12) Å, V = 1489.77(19) Å^3^, Z = 4, T = 100 K, μ(MoKα) = 0.091 mm^–1^, D_calc._ = 1.286 g/cm^3^, 37377 measured reflections, 2650 independent reflections (R_int_ = 0.074). Final R indices R1 = 0.0386 [for 2468 reflections, with I > 2σ(I)], wR2 = 0.0924 (all data).

### 4.15. Synthesis of (8S*,9S*,12R*)-9-Hydroxy-10-methylidene-8-propyl-5,13-dioxatricyclo[10.3.0.0^3,7^]penta-deca-1(15),3,6-trien-14-one and (8S*,9R*,12R*)-9-Hydroxy-10-methylidene-8-propyl-5,13-dioxatricyclo-[10.3.0.0^3,7^]pentadeca-1(15),3,6-trien-14-one *(**16c**, **16d**)*

To a solution of the alcohol **15b** (59.2 mg, 0.142 mmol) in CH_2_Cl_2_ (2.5 mL) was added Dess-Martin periodinane (80.2 mg, 0.189 mmol, 1.54 equiv.). The mixture was stirred at room temperature for 40 min, cooled to 0 °C and the reaction was quenched with a saturated aqueous solution of Na_2_S_2_O_3_ (5 mL). The mixture was diluted with water (5 mL) and CH_2_Cl_2_ (5 mL) and after 10 min, the phases were separated. The aqueous phase was extracted with CH_2_Cl_2_ (3 × 5 mL) and the combined organic extracts were washed with brine (10 mL), dried with Na_2_SO_4_, filtered and concentrated in vacuo. The crude product was purified quickly by passage through a small plug of silica (PE-Et_2_O, 4:1) to afford the corresponding aldehyde (50.9 mg, 100%) as a colourless oil.

To a solution of CrCl_2_ (215 mg, 1.75 mmol, 12.4 equiv.) and NiCl_2_ (2.5 mg, 0.019 mmol, 13 mol %) in degassed (three freeze-thaw cycles) DMSO (6 mL) was added a solution of aldehyde (50.9 mg) in degassed (three freeze-thaw cycles) DMSO (6.5 mL) at room temperature. The dark green mixture was stirred for 48 h at 50 °C. The reaction was quenched with saturated aqueous NH_4_Cl (15 mL) and the mixture diluted with EtOAc (30 mL). The biphasic mixture was stirred for 30 min and the phases were separated. The aqueous phase was extracted with EtOAc (3 × 30 mL) and the combined organic extracts were washed with brine (50 mL), dried over MgSO_4_, filtered and concentrated in vacuo. The crude product was purified by flash column chromatography (PE-Et_2_O, 9:11) to give an inseparable mixture (1:2.4 / 2.4:1 based on ^1^H-NMR analysis) of the diastereoisomeric alcohols **16c** and **16d** (17.8 mg, 43%). R*_f_* = 0.25 (PE-Et_2_O, 2:3); IR ν_max_ 3448, 2957, 2928, 2872, 2360, 1748, 1633, 1541, 1465 cm^−1^; *Minor diastereomer* ^1^H-NMR (500 MHz, CDCl_3_) δ 7.34 (1H, d, *J* = 1.4 Hz), 7.32 (1H, d, *J* = 1.4 Hz), 6.02 (1H, br s), 5.14 (1H, s), 5.05 (1H, s), 4.98 (1H, ddd, *J* = 4.5, 3.6, 1.0 Hz), 4.14 (1H, s), 3.76 (1H, dd, *J* = 18.0, 0.8 Hz), 3.67 (1H, d, *J* = 18.0 Hz), 2.60−2.48 (2H, m), 2.05 (1H, app ddd, *J* = 15.2, 3.6, 1.3 Hz), 2.11 (1H, br s), 1.92 (1H, br s), 1.56−1.49 (1H, m), 1.37−1.24 (1H, m), 1.23−1.11 (1H, m), 0.87 (3H, t, *J* = 7.4 Hz); ^13^C-NMR (126 MHz, CDCl_3_) δ 172.6, 171.4, 146.4, 142.1, 140.4, 123.7, 119.5, 119.4, 119.2, 82.6, 77.8, 39.6, 35.2, 30.9, 24.2, 20.7, 14.0; Major diastereomer ^1^H-NMR (500 MHz, CDCl_3_) δ 7.28 (1H, s), 7.23 (1H, d, *J* = 1.5 Hz), 5.90 (1H, ddd, *J* = 1.5, 1.5, 1.5 Hz), 5.13 (1H, s), 5.05 (1H, s), 4.75 (1H, br s), 3.98 (1H, d, *J* = 7.4 Hz), 3.83−3.73 (1H, m), 3.61 (1H, d, *J* = 17.9 Hz), 2.60−2.48 (2H, m), 2.39 (1H, d, *J* = 15.4 Hz), 1.92 (1H, br s), 1.78−1.61 (2H, m), 1.37−1.24 (1H, m), 1.23−1.11 (1H, m), 0.87 (3H, t, *J* = 7.4 Hz); ^13^C-NMR (126 MHz, CDCl_3_) δ 172.5, 168.6, 143.4, 141.2, 140.4, 123.7, 119.1, 116.9, 116.4, 86.2, 81.5, 40.0, 37.1, 31.1, 22.5, 20.8, 14.2; LRMS (CI, isobutane): *m*/*z* (int) 289 (100), 271 (24), 137 (10), 71 (13). HRMS (CI, isobutane) calculated for C_17_H_21_O_4_ [M + H]^+^: 289.1440; found 289.1436.

### 4.16. Synthesis of (8S*,12R*)-10-Methylidene-8-propyl-5,13-dioxatricyclo[10.3.0.0^3,7^]pentadeca-1(15),3,6-triene-9,14-dione *(**17**)*

To a solution of the diastereoisomeric alcohols **16c** and **16d** (7.1 mg, 0.025 mmol) in CH_2_Cl_2_ (1 mL) was added Dess-Martin periodinane (15.6 mg, 0.0368 mmol, 1.49 equiv.). The solution was stirred at room temperature for 1 h and then cooled to 0 °C. The reaction was quenched by the addition of a saturated aqueous solution of Na_2_S_2_O_3_ (5 mL) and the mixture was diluted with water (5 mL) and CH_2_Cl_2_ (5 mL). After 10 min, the phases were separated and the aqueous phase was extracted with CH_2_Cl_2_ (3 × 5 mL). The organic extracts were combined and washed with brine (10 mL), dried with Na_2_SO_4_, filtered and concentrated in vacuo. The crude product was purified by passage through a small plug of silica (PE-Et_2_O, 5:5) to give the enone **17** (7.0 mg, 99%) of a colourless oil. R*_f_* = 0.50 (PE-Et_2_O, 1:4); IR ν_max_ 3154, 3100, 2959, 2933, 2922, 2872, 1791, 1755, 1688, 1634, 1534, 981, 923, 898, 875, 859, 804, 765, 746 cm^−1^; ^1^H-NMR (500 MHz, CDCl_3_) δ 7.29 (1H, d, *J* = 1.2 Hz), 7.28 (1H, s), 5.88 (1H, td, *J* = 1.9, 1.3 Hz), 5.74 (1H, d, *J* = 1.2 Hz), 5.70 (1H, d, *J* = 1.2 Hz), 4.81 (1H, ddd, *J* = 8.6, 5.2, 1.3 Hz), 4.09 (1H, ddd, *J* = 10.3, 4.2, 1.2 Hz), 3.62 (2H, d, *J* = 1.9 Hz), 3.46 (1H, dd, *J* = 13.9, 5.2 Hz), 2.42 (1H, dd, *J* = 13.9, 8.6 Hz), 2.19−2.12 (1H, m), 1.75 (1H, dddd, *J* = 13.0, 8.5, 7.5, 4.2 Hz), 1.39 (2H, app tq, *J* = 7.5, 7.3 Hz), 0.99 (3H, t, *J* = 7.3 Hz).

### 4.17. Luche Reduction of (8S*,12R*)-10-Methylidene-8-propyl-5,13-dioxatricyclo[10.3.0.0^3,7^]pentadeca-1(15), 3,6-triene-9,14-dione *(**17**)*

To a solution of the enone **17** (5.3 mg, 19 μmol) in MeOH (1.8 mL) was added CeCl_3_·7H_2_O (24 mg, 64 μmol, 3.4 equiv.). The reaction was cooled to −78 °C before addition of NaBH_4_ (1.6 mg, 42 μmol, 2.2 equiv.). The mixture was stirred for 1 h and the reaction was quenched by the addition of saturated aqueous NH_4_Cl (5 mL) and water (5mL). The mixture was warmed to room temperature and EtOAc (10 mL) was added. The phases were separated and the aqueous phase was extracted with EtOAc (3 × 10 mL). The organic extracts were combined, washed with brine (20 mL), dried over MgSO_4_, filtered and concentrated in vacuo. Analysis of the residue (5.3 mg, quant.) by ^1^H-NMR revealed a mixture of diastereoisomeric alcohols **16c** and **16d** with a slightly higher ratio (1:3 / 3:1 determined by ^1^H-NMR analysis) in favour of the major isomer produced by the original NHK reaction.

## Supplementary Materials

The following are available online at https://www.mdpi.com/1420-3049/24/14/2654/s1, ^1^H and ^13^C-NMR spectra for key compounds **4**, **12**, **15a**, **15b**, **16a**–**d**.

## References

[1] Haneishi T., Nakajima M., Koi K., Furuya K., Iwado S., Sato S. (1990). Anhydride Herbicides. U.S. Patent.

[2] Haneishi T., Nakajima M., Koi K., Furuya K., Iwado S., Sata S. (1991). Herbicide, Its Preparation and Use. U.S. Patent.

[3] Nakajima M., Itoi K., Takamatsu Y., Sato S., Furukawa Y., Furuya K., Honma T., Kadotani J., Kozasa M., Haneishi T. (1991). Cornexistin: A New Fungal Metabolite with Herbicidal Activity. J. Antibiot..

[4] Fields S.C., Gerwick B.C., Mireles-Lo L. (1995). Hydroxycornexistin. U.S. Patent.

[5] Fields S.C., Mireles-Lo L., Gerwick B.C. (1996). Hydroxycornexistin: A New Phytotoxin from *Paecilomyces Variotii*. J. Nat. Prod..

[6] Mioso R., Toledo Marante F.J., Herrera Bravo de Laguna I. (2015). The Chemical Diversity of the Ascomycete Fungus *Paecilomyces Variotii*. Appl. Biochem. Biotechnol..

[7] Chen X., Zheng Y., Shen Y. (2007). Natural Products with Maleic Anhydride Structure: Nonadrides, Tautomycin, Chaetomellic Anhydride, and Other Compounds. Chem. Rev..

[8] Amagasa T., Paul R.N., Heitholt J.J., Duke S.O. (1994). Physiological Effects of Cornexistin on *Lemna pausicostata*. Pestic. Biochem. Physiol..

[9] Clark J.S., Marlin F., Nay B., Wilson C. (2003). Synthesis of the Carbocyclic Core of the Cornexistins by Ring-Closing Metathesis. Org. Lett..

[10] Clark J.S., Northall J.M., Marlin F., Nay B., Wilson C., Blake A.J., Waring M.J. (2008). Synthetic Studies on the Cornexistins: Synthesis of (±)-5-*epi*-Hydroxycornexistin. Org. Biomol. Chem..

[11] Tung J.C., Chen W., Noll B.C., Taylor R.E., Fields S.C., Dent W.H., Green F.R. (2007). Towards the Synthesis of the Cornexistins. Synthesis.

[12] Tian Q., Zhang G. (2016). Recent Advances in the Asymmetric Nozaki-Hiyama-Kishi Reaction. Synthesis.

[13] Aimon A. (2013). A NHK Approach Towards the Total Synthesis of the Cornexistins. Ph.D. Thesis.

[14] Fumiyama H., Sadayuki T., Osada Y., Goto Y., Nakao Y., Hosokawa S. (2016). Synthesis and Antileishmanial Activity of the Core Structure of Cristaxenicin A. Bioorg. Med. Chem. Lett..

[15] Momose T., Toyooka N., Nishi T., Takeuchj Y. (1988). 2(3*H*)- and 2(5*H*)-Furanones. III. An Efficient Synthesis and the Eschenmoser-Mannich Reaction of *N*-Substituted 4-Amino-2(5*H*)-furanones. Heterocycles.

[16] Marshall J.A., Jenson T.M., DeHoff B.S. (1986). [2,3]-Wittig Ring Contraction. A New Route to Cembranoid Natural Products. J. Org. Chem..

[17] Wulff W.D., Peterson G.A., Bauta W.E., Chan K.-S., Faron K.L., Gilbertson S.R., Kaesler R.W., Yang D.C., Murray C.K. (1986). A Regioselective Entry to Vinyl Lithiums from Unsymmetrical Ketones via Enol Triflates. J. Org. Chem..

[18] Cook M.J., Forbes E.J. (1968). [4,5-c] Furotropone: A New Heterocyclic System. Tetrahedron.

[19] Tanis S.P., Head D.B. (1984). Furans in Synthesis 4. Silyl Furans as Butenolide Equivalents. Tetrahedron Lett..

[20] Farina V., Krishnan B. (1991). Large Rate Accelerations in the Stille Reaction with Tri-2-furylphosphine and Triphenylarsine as Palladium Ligands: Mechanistic and Synthetic Implications. J. Am. Chem. Soc..

[21] Smith N.D., Mancuso J., Lautens M. (2000). Metal-Catalyzed Hydrostannations. Chem. Rev..

[22] The X-ray data for the alcohol **16a** have been deposited in the Cambridge Crystallographic Database (CCDC 1920025). http://www.ccdc.cam.ac.uk/conts/retrieving.html.

