# Supplementary material for: Synthesis of the Core Framework of the Cornexistins by Intramolecular Nozaki-Hiyama-Kishi Coupling
Source: Molecules. 2019 Jul 22;24(14):2654. doi: 10.3390/molecules24142654 (PMC6680490; doi:10.3390/molecules24142654)
Supplement: Supplementary file 1 [file molecules-24-02654-s001.pdf]

Synthesis the Core Framework of the Cornexistins by Intramolecular Nozaki-Hiyama-Kishi Coupling

Anthony Aimon, Louis J. Farrugia and J. Stephen Clark*

School of Chemistry, Joseph Black Building, University of Glasgow,
University Avenue, Glasgow, G12 8QQ, United Kingdom

Supporting Information

^{1}H and ^{13}C NMR Spectra for Key Compounds

	Page
^{1}H NMR spectrum of **4**	2
^{13}C NMR spectrum of **4**	3
^{1}H NMR spectrum of **12**	4
^{13}C NMR spectrum of **12**	5
^{1}H NMR spectrum of **15a**	6
^{13}C NMR spectrum of **15a**	7
^{1}H NMR spectrum of **15b**	8
^{13}C NMR spectrum of **15b**	9
^{1}H NMR spectrum of **16a**	10
^{13}C NMR spectrum of **16a**	11
^{1}H NMR spectrum of **16b**	12
^{13}C NMR spectrum of **16b**	13
^{1}H NMR spectrum of **16c,d**	14
^{13}C NMR spectrum of **16c,d**	15
^{1}H NMR spectrum of **17**	16

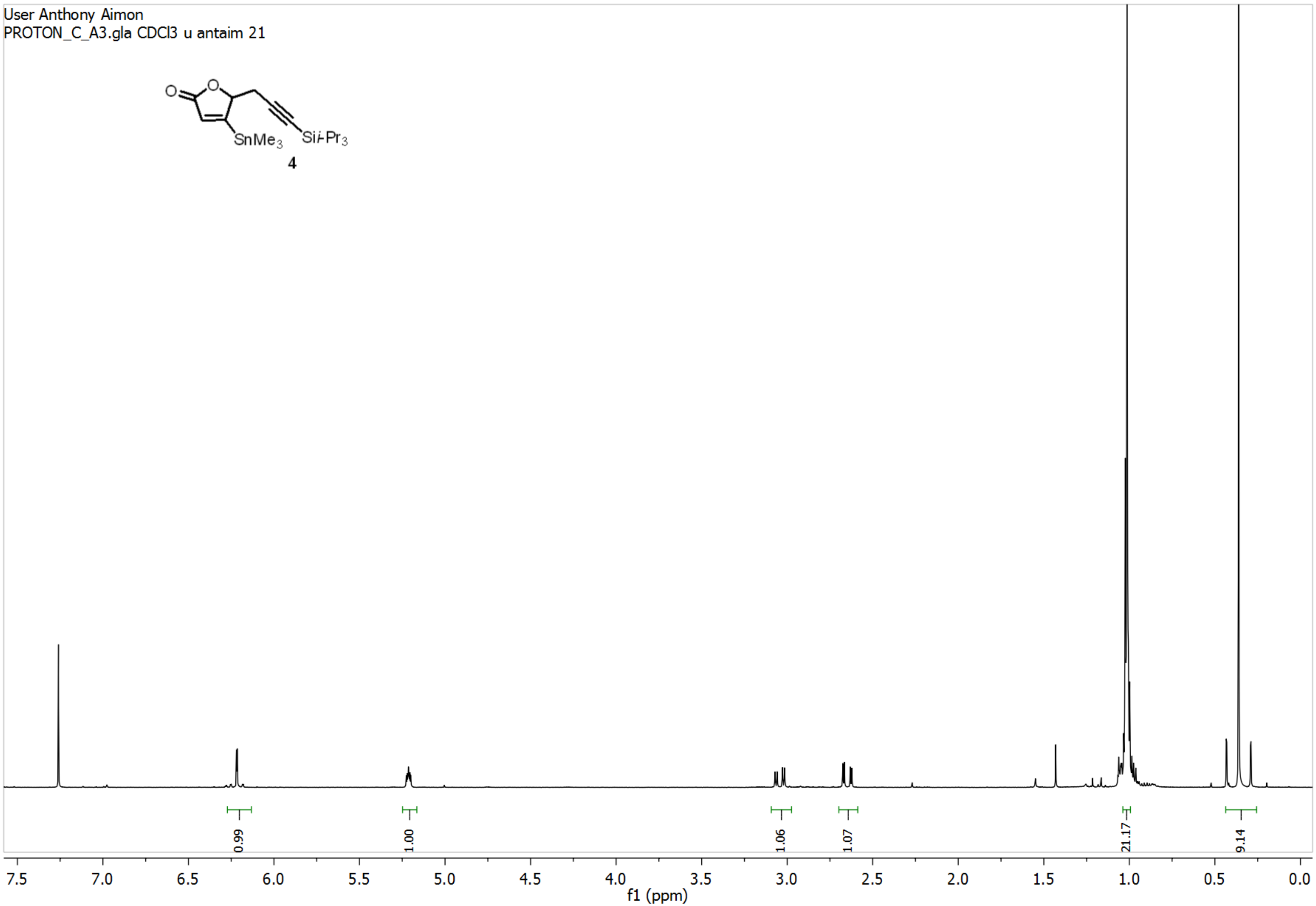
User Anthony Aimon
PROTON_C_A3.gla CDCl3 u antaim 21
O
O
SnMe3
Si*i*-Pr3
4
0.99
1.00
1.06
1.07
21.17
9.14
7.5
7.0
6.5
6.0
5.5
5.0
4.5
4.0
3.5
3.0
2.5
2.0
1.5
1.0
0.5
0.0
f1 (ppm)

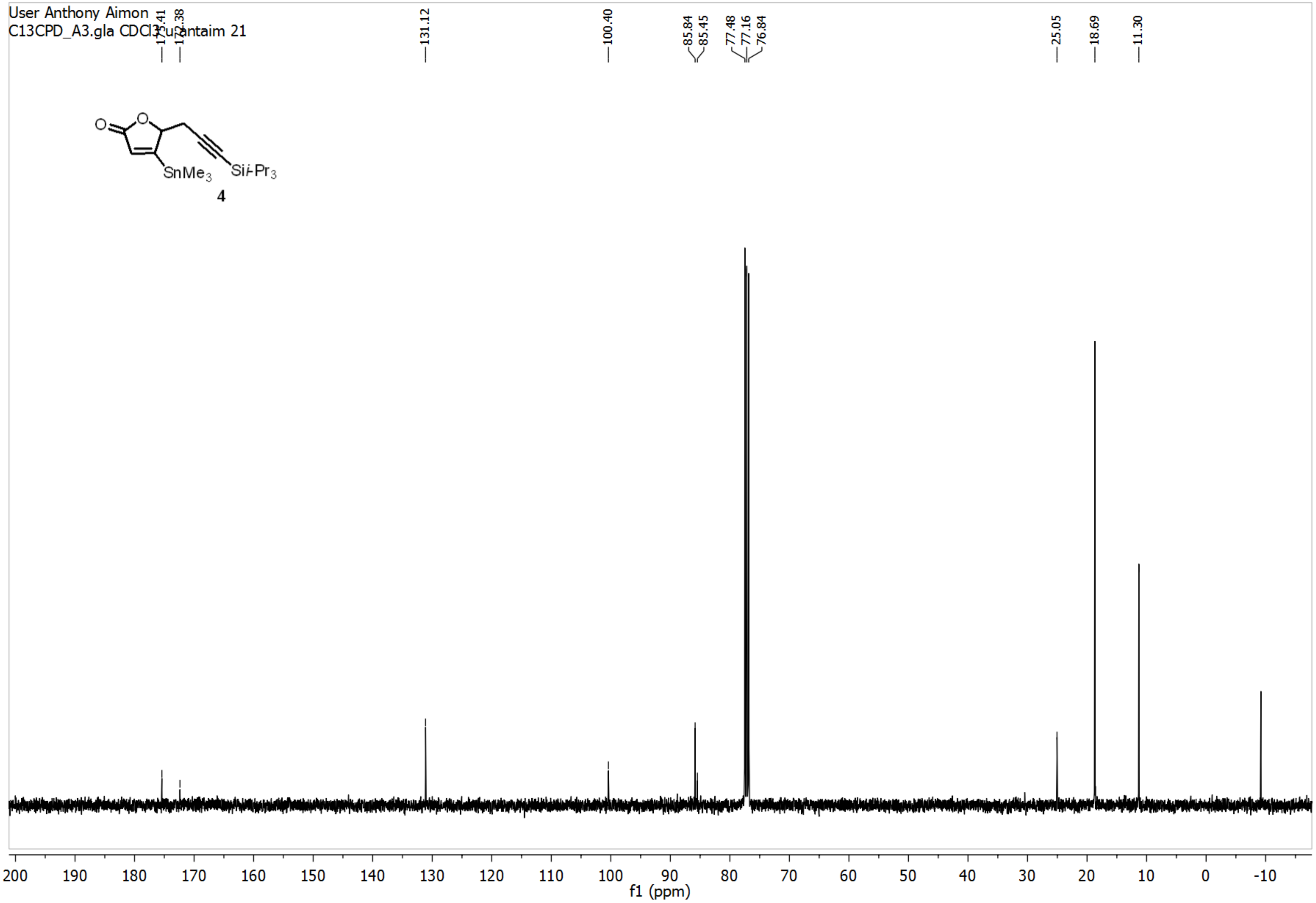
User Anthony Aimon
131.12
100.40
85.84
85.45
77.48
77.16
76.84
25.05
18.69
11.30
O
O
SnMe3
Si*i*-Pr3
4
f1 (ppm)

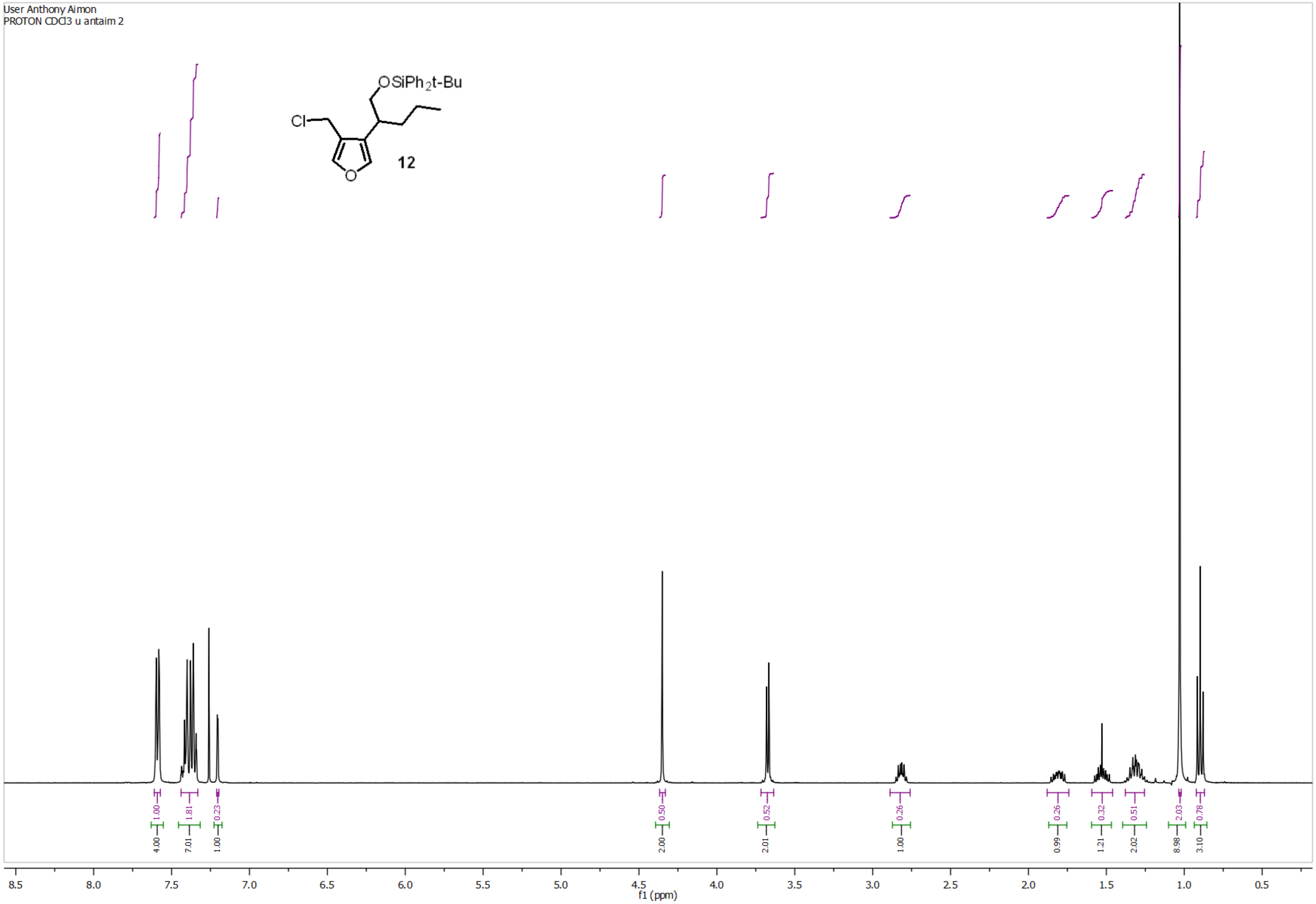

User Anthony Aimon
PROTON CDCl3 u antaim 2
OSiPh2t-Bu
Cl
O
12
f1 (ppm)

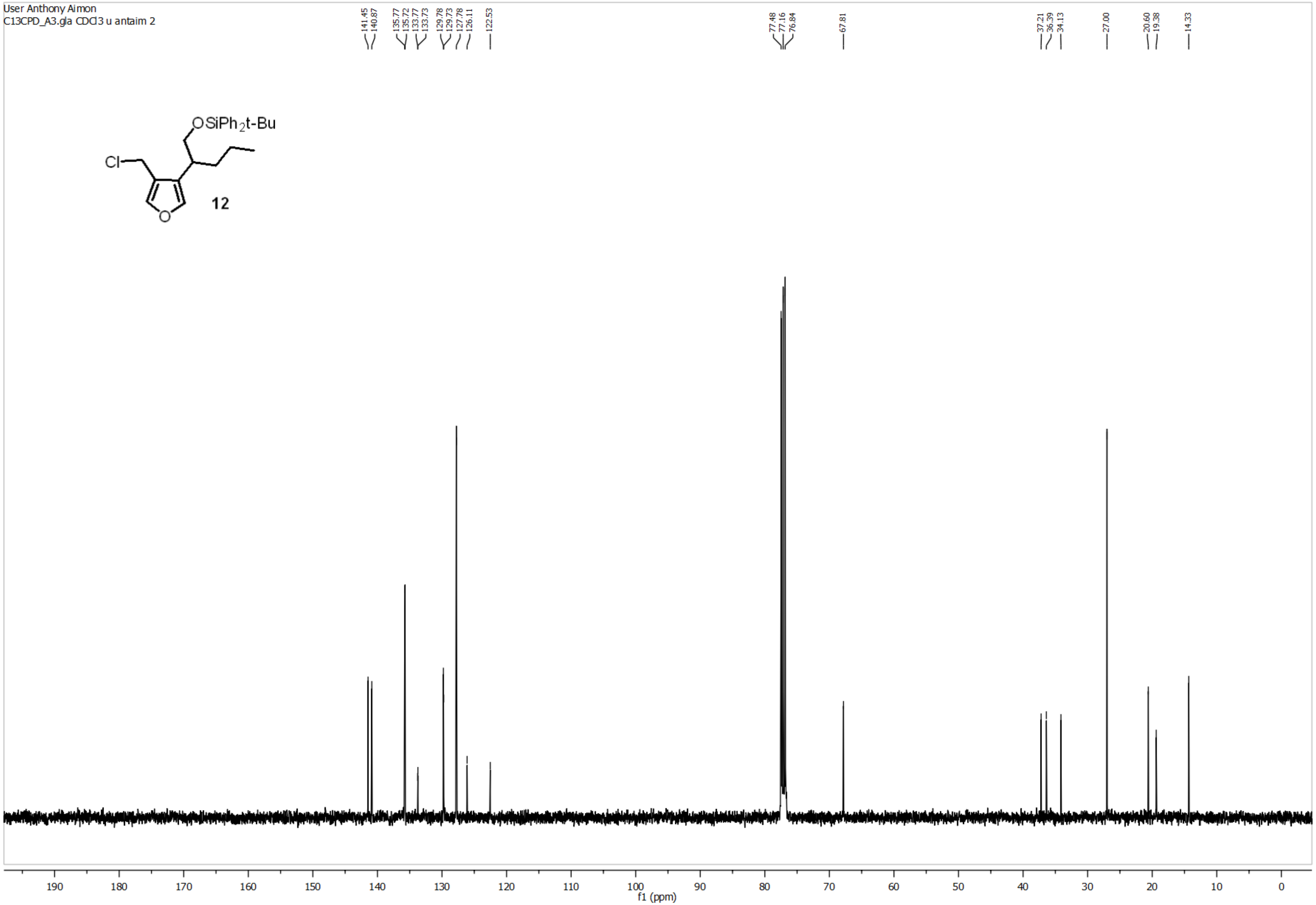
User Anthony Aimon
C13CPD_A3.gla CDCl3 u antaim 2
141.45
140.87
135.77
135.72
133.77
133.73
129.78
129.73
127.78
126.11
122.53
77.48
77.16
76.84
67.81
37.21
36.39
34.13
27.00
20.60
19.38
14.33
OSiPh2t-Bu
Cl
O
12
f1 (ppm)

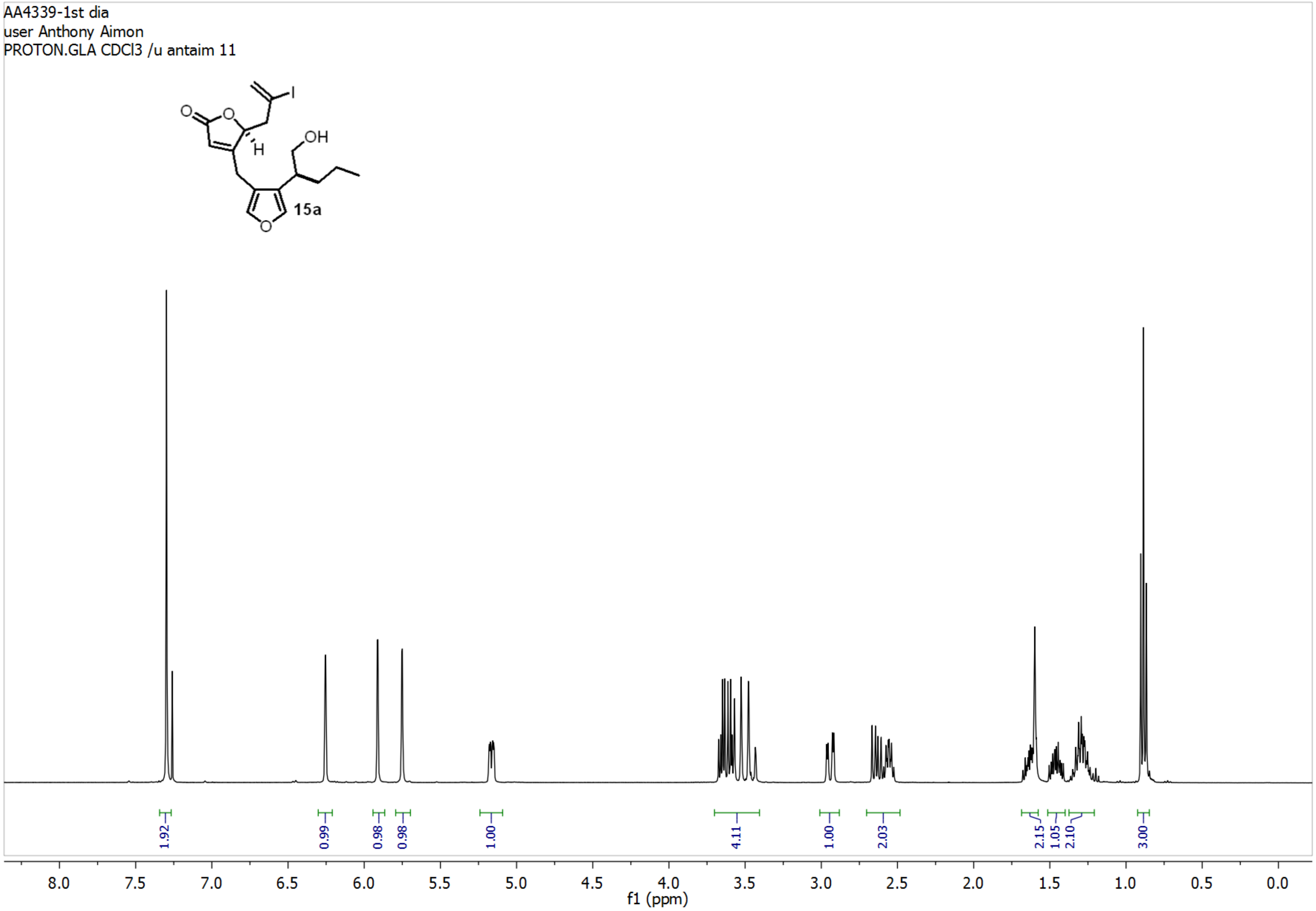
AA4339-1st dia
user Anthony Aimon
PROTON.GLA CDCl3 /u antaim 11
O
O
I
OH
H
O
15a
1.92
0.99
0.98
0.98
1.00
4.11
1.00
2.03
2.15
1.05
2.10
3.00
8.0
7.5
7.0
6.5
6.0
5.5
5.0
4.5
4.0
3.5
3.0
2.5
2.0
1.5
1.0
0.5
0.0
f1 (ppm)

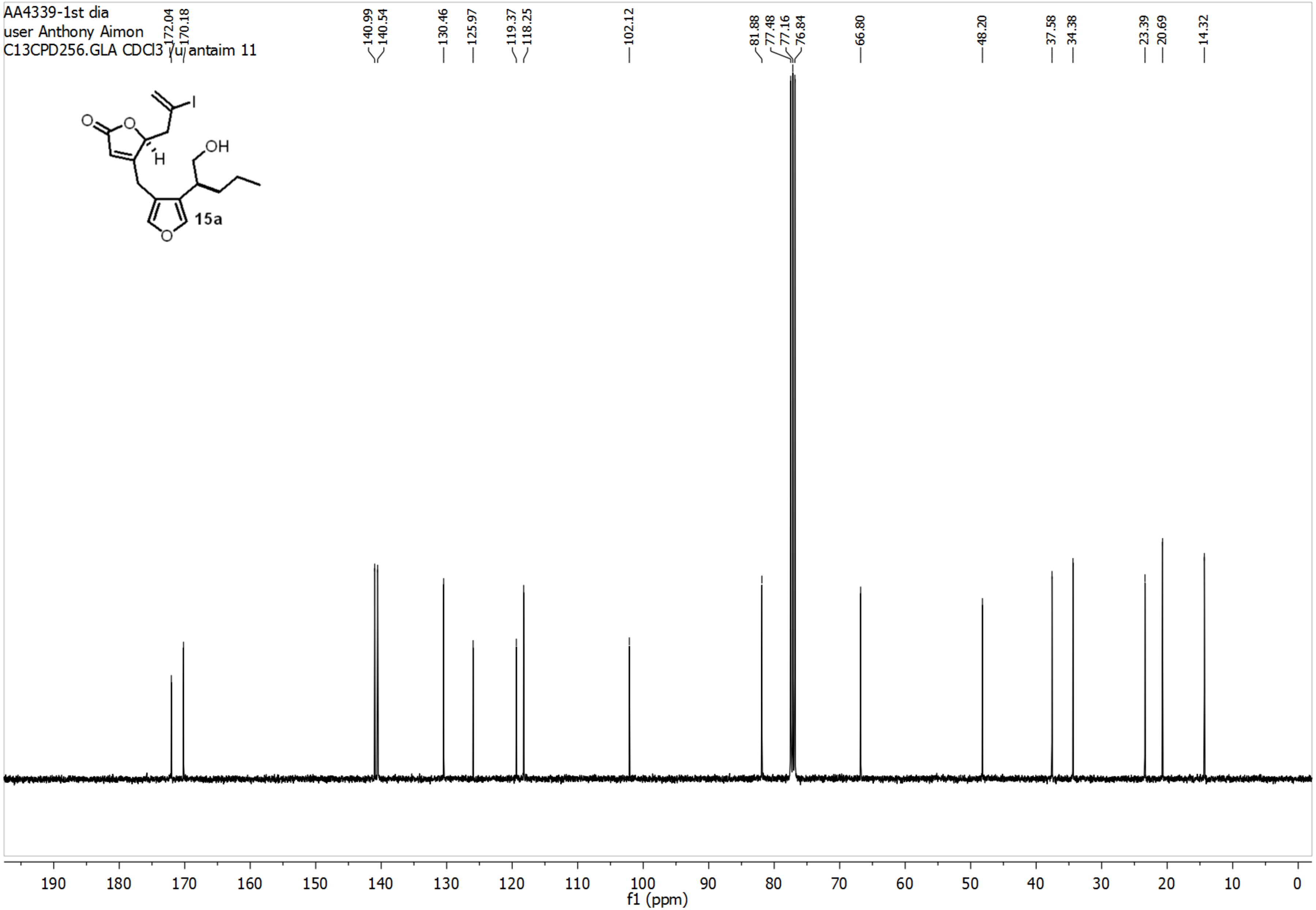
AA4339-1st dia
user Anthony Aimon
C13CPD256.GLA CDCl3 /u antaim 11
172.04
170.18
140.99
140.54
130.46
125.97
119.37
118.25
102.12
81.88
77.48
77.16
76.84
66.80
48.20
37.58
34.38
23.39
20.69
14.32
15a
OH
H
O
I
190 180 170 160 150 140 130 120 110 100 90 80 70 60 50 40 30 20 10 0
f1 (ppm)

AA4340-2nd dia
user Anthony Aimon
PROTON.GLA CDCl3 /u antaim 12

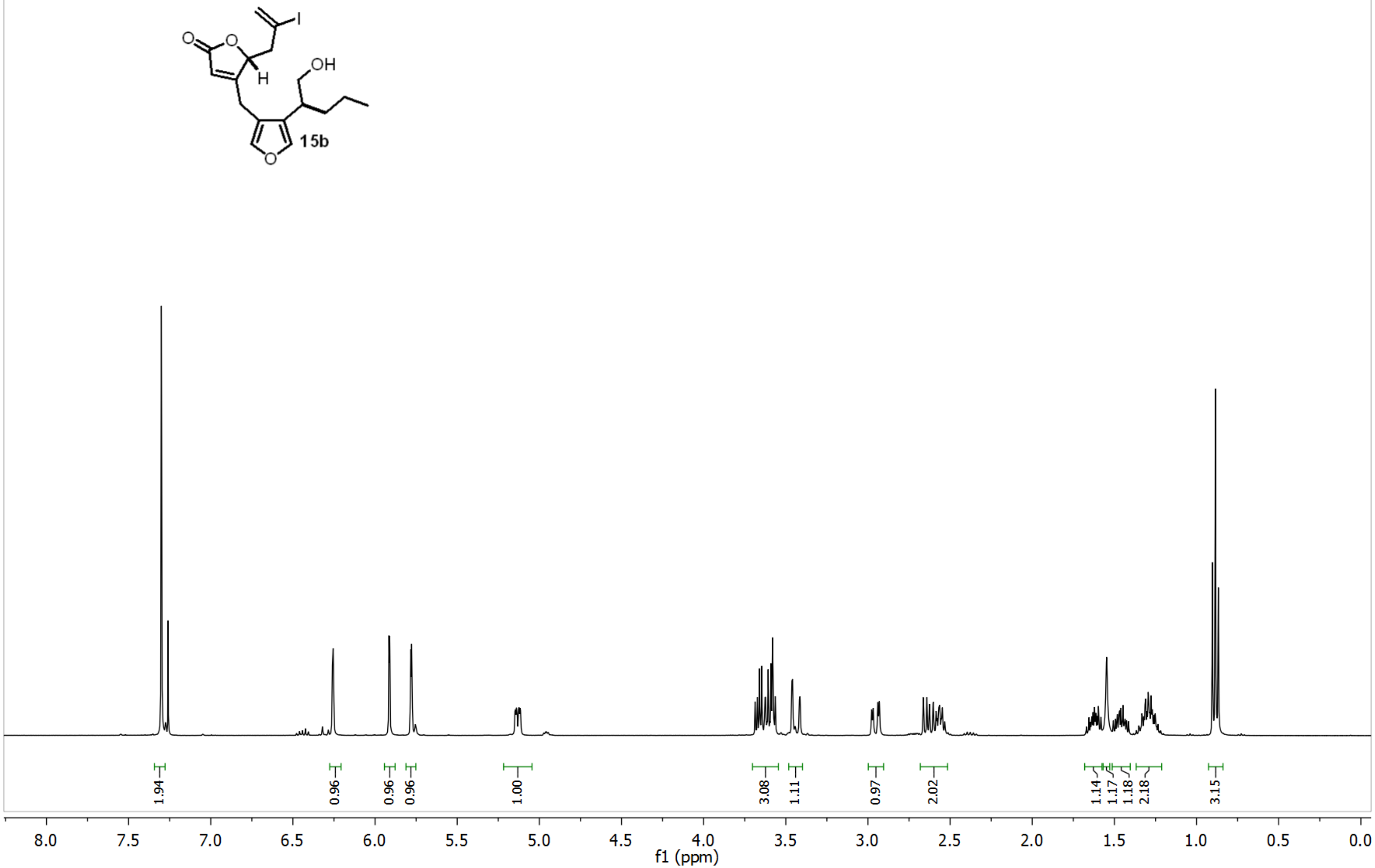

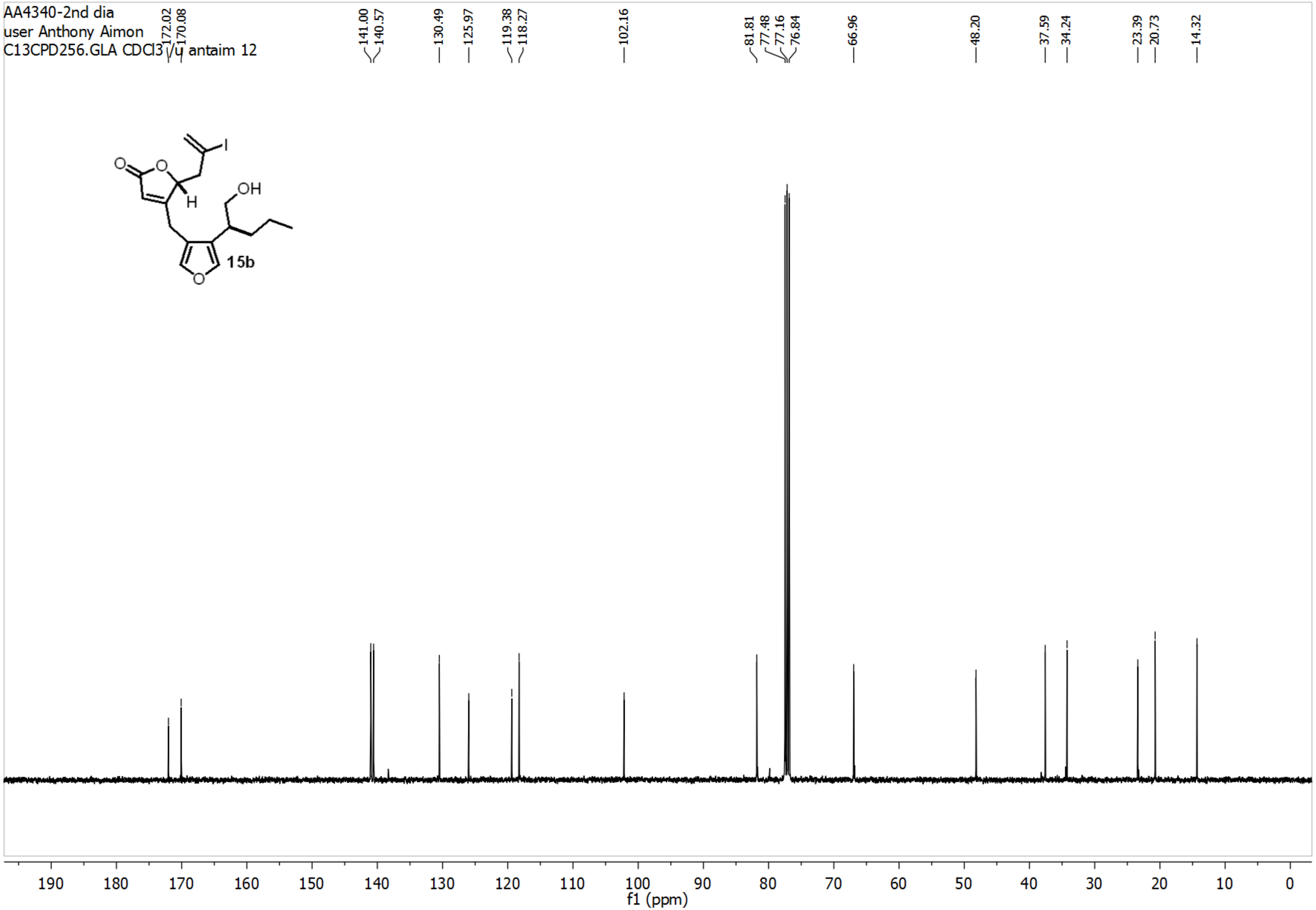
AA4340-2nd dia
user Anthony Aimon
C13CPD256.GLA CDCl3 /u antaim 12
172.02
170.08
141.00
140.57
130.49
125.97
119.38
118.27
102.16
81.81
77.48
77.16
76.84
66.96
48.20
37.59
34.24
23.39
20.73
14.32
OH
O
H
I
15b
190
180
170
160
150
140
130
120
110
100
90
80
70
60
50
40
30
20
10
0
f1 (ppm)

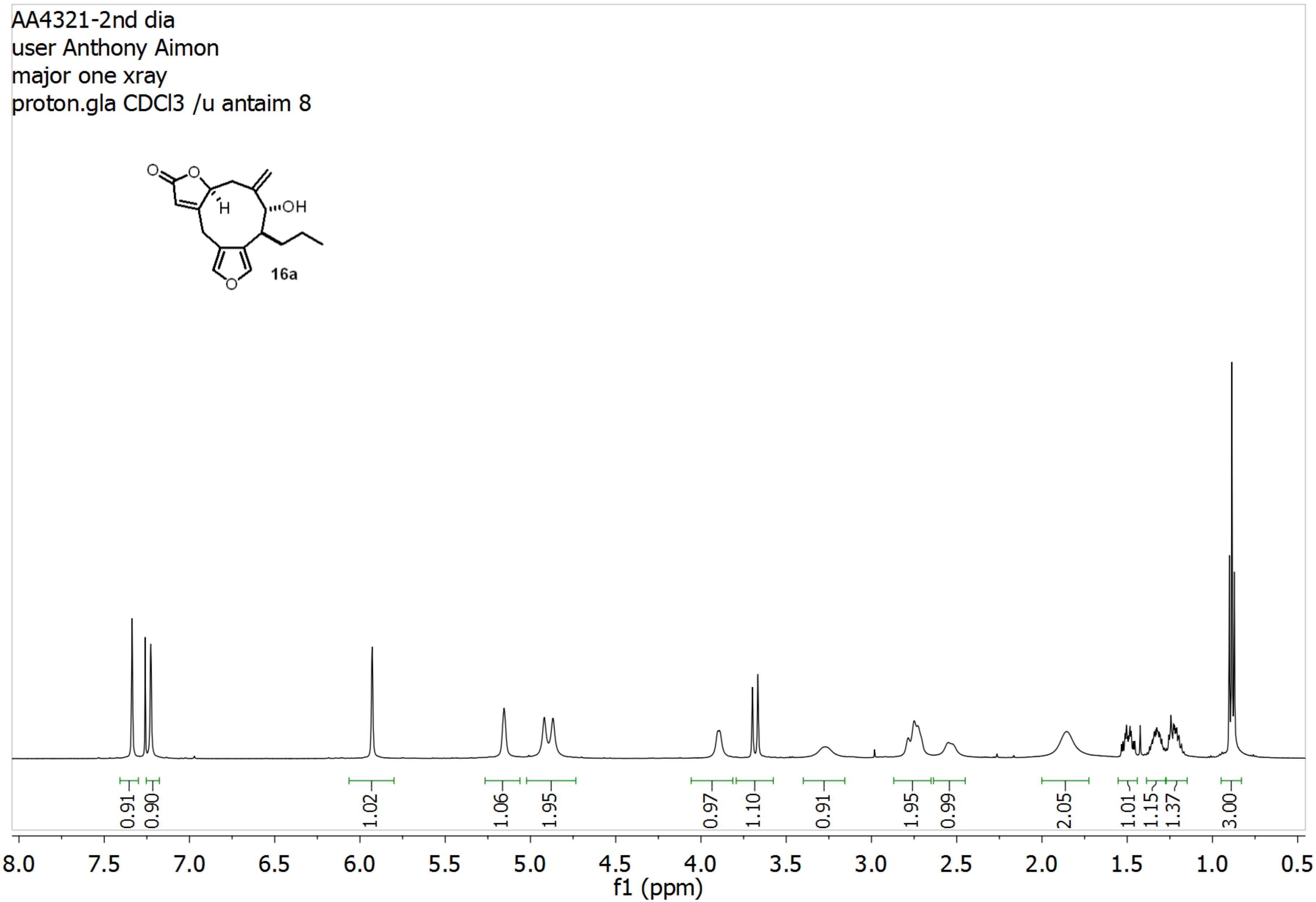
AA4321-2nd dia
user Anthony Aimon
major one xray
proton.gla CDCl3 /u antaim 8
16a
OH
H
0.91
0.90
1.02
1.06
1.95
0.97
1.10
0.91
1.95
0.99
2.05
1.01
1.15
1.37
3.00
8.0 7.5 7.0 6.5 6.0 5.5 5.0 4.5 4.0 3.5 3.0 2.5 2.0 1.5 1.0 0.5
f1 (ppm)

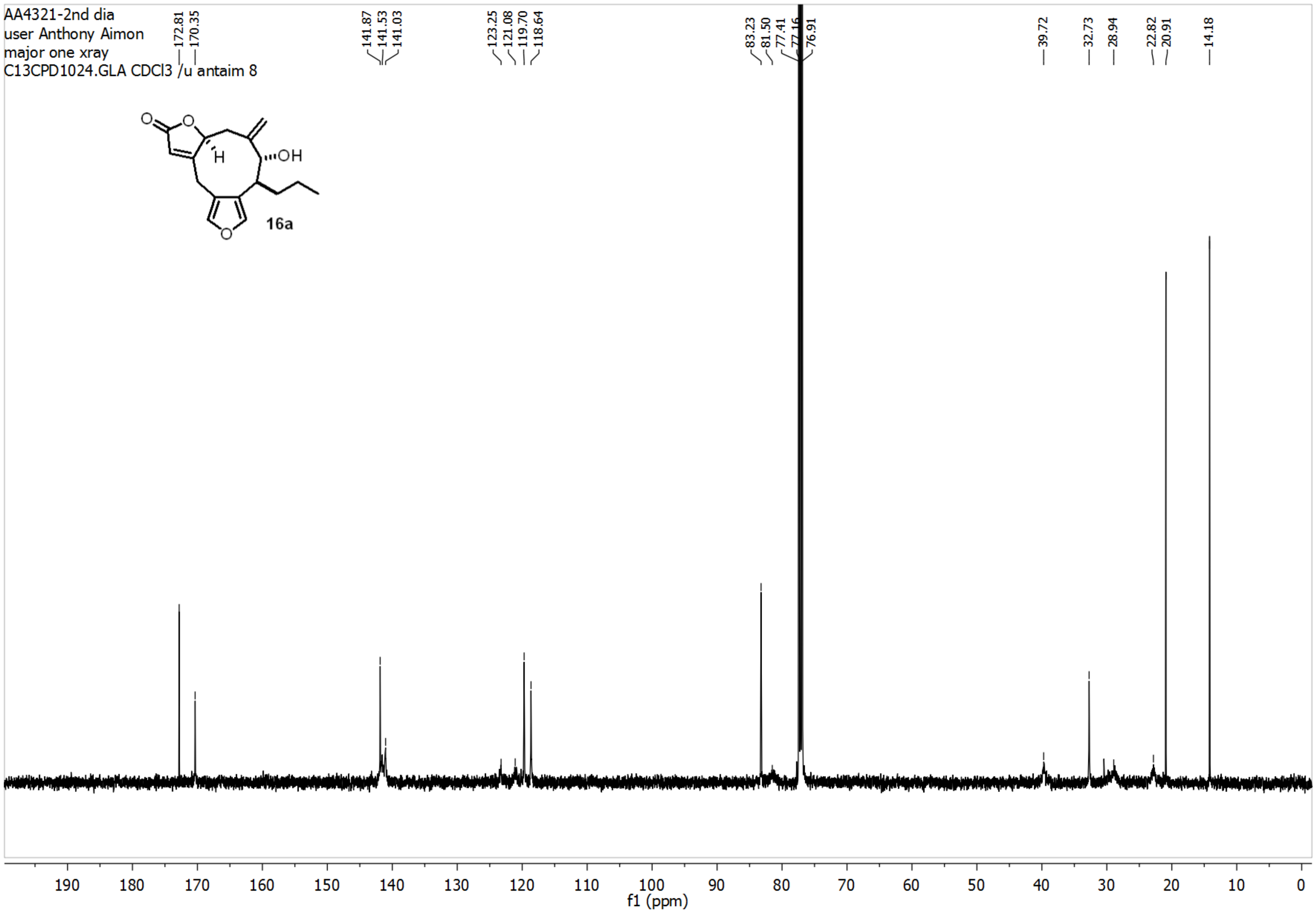
AA4321-2nd dia
user Anthony Aimon
major one xray
C13CPD1024.GLA CDCl3 /u antaim 8
172.81
170.35
141.87
141.53
141.03
123.25
121.08
119.70
118.64
83.23
81.50
77.41
77.16
76.91
39.72
32.73
28.94
22.82
20.91
14.18
16a
190 180 170 160 150 140 130 120 110 100 90 80 70 60 50 40 30 20 10 0
f1 (ppm)

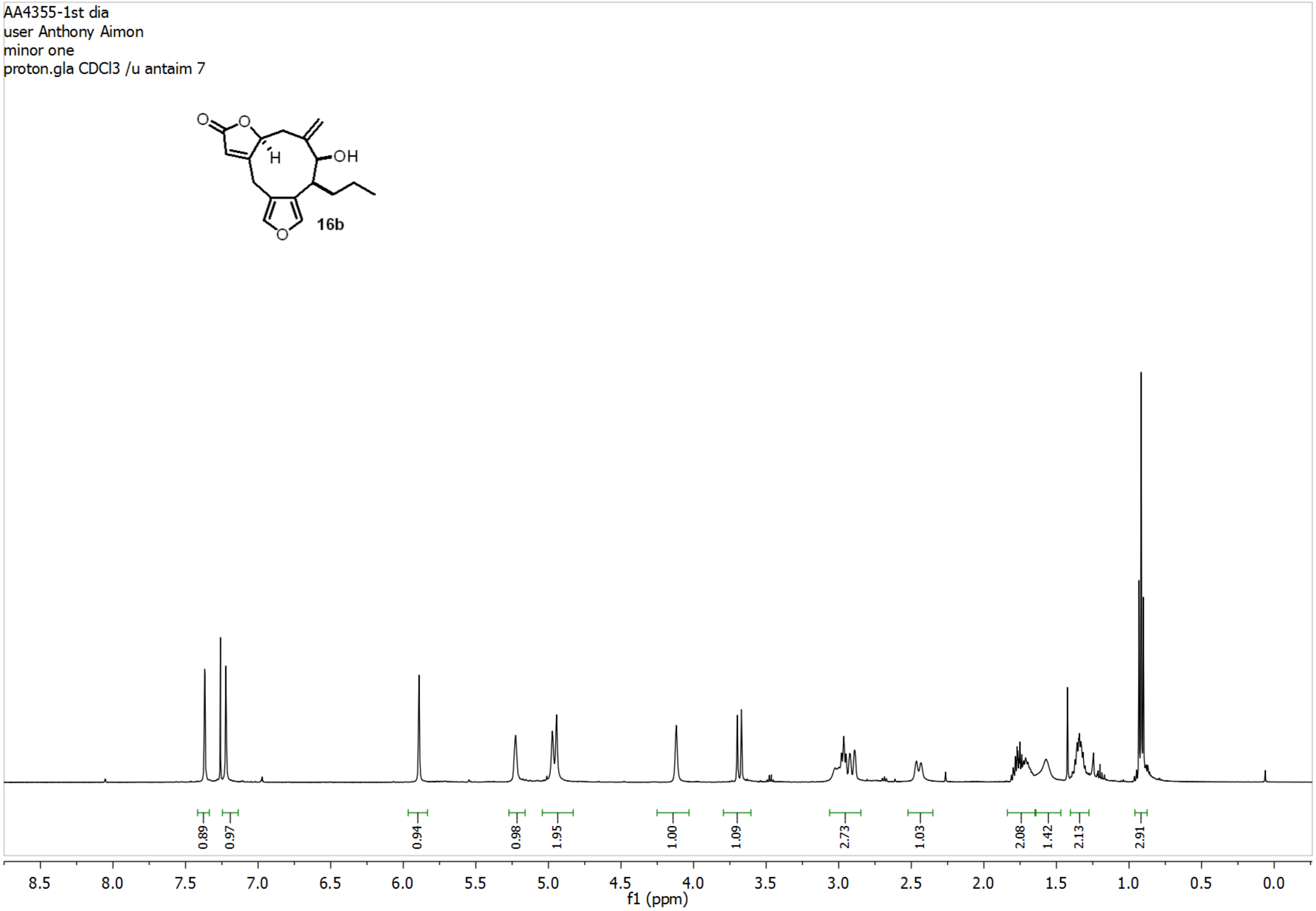
AA4355-1st dia
user Anthony Aimon
minor one
proton.gla CDCl3 /u antaim 7
O
O
H
OH
O
16b
0.89
0.97
0.94
0.98
1.95
1.00
1.09
2.73
1.03
2.08
1.42
2.13
2.91
8.5
8.0
7.5
7.0
6.5
6.0
5.5
5.0
4.5
4.0
3.5
3.0
2.5
2.0
1.5
1.0
0.5
0.0
f1 (ppm)

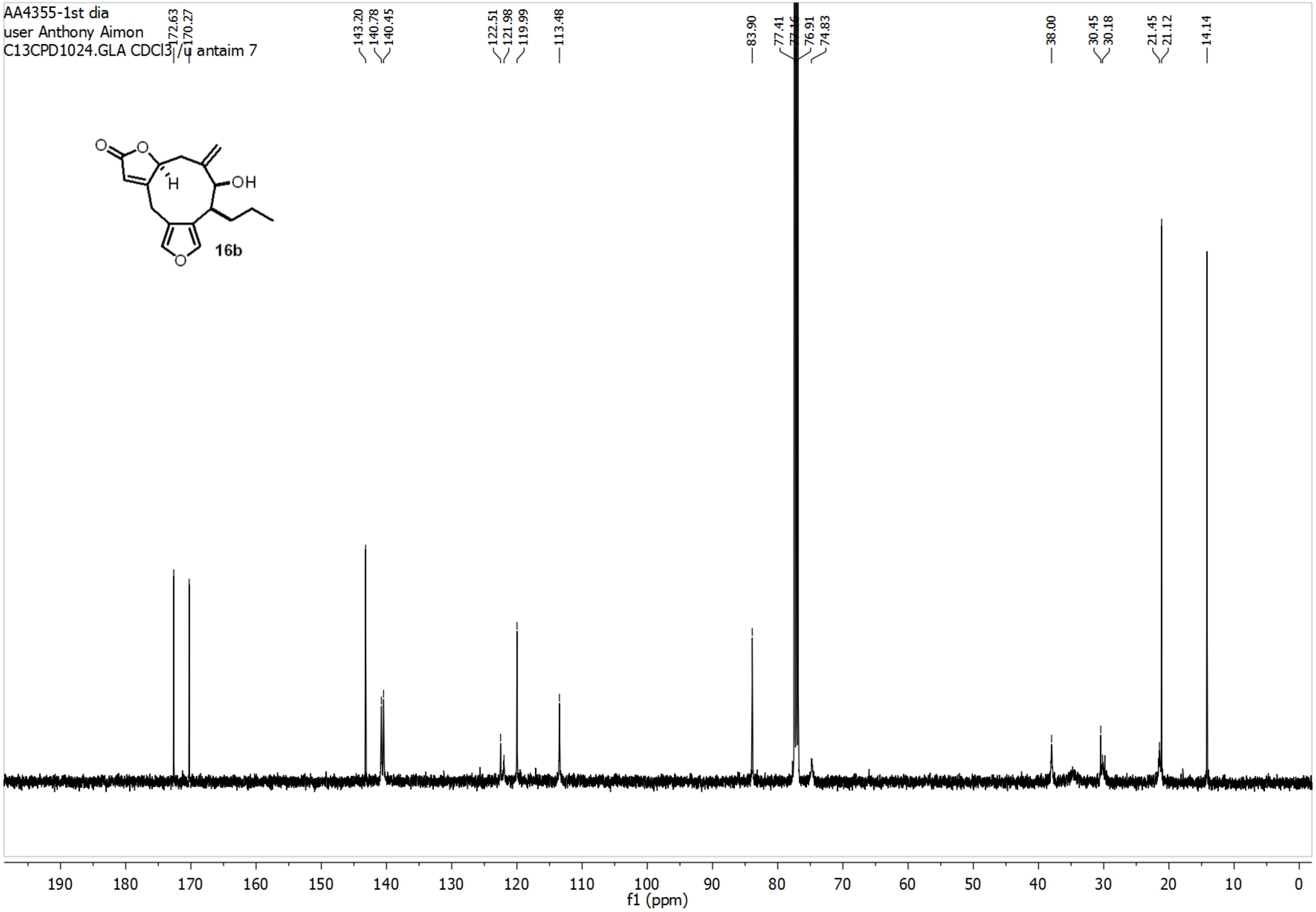
AA4355-1st dia
user Anthony Aimon
C13CPD1024.GLA CDCl3 /u antaim 7
172.63
170.27
143.20
140.78
140.45
122.51
121.98
119.99
113.48
83.90
77.41
76.91
74.83
38.00
30.45
30.18
21.45
21.12
14.14
OH
H
O
16b
f1 (ppm)

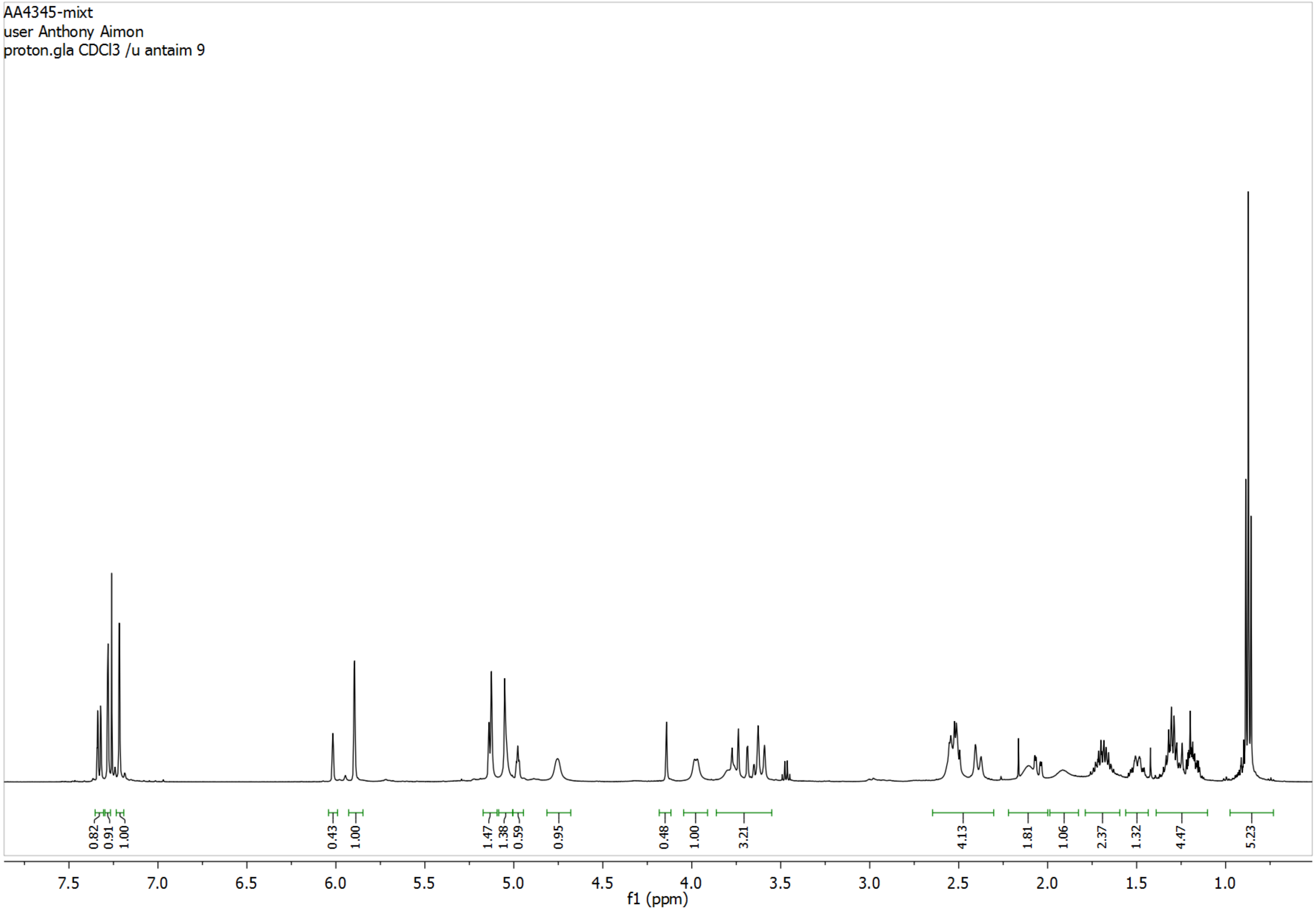
AA4345-mixt
user Anthony Aimon
proton.gla CDCl3 /u antaim 9
0.82
0.91
1.00
0.43
1.00
1.47
1.38
0.59
0.95
0.48
1.00
3.21
4.13
1.81
1.06
2.37
1.32
4.47
5.23
7.5
7.0
6.5
6.0
5.5
5.0
4.5
4.0
3.5
3.0
2.5
2.0
1.5
1.0
f1 (ppm)

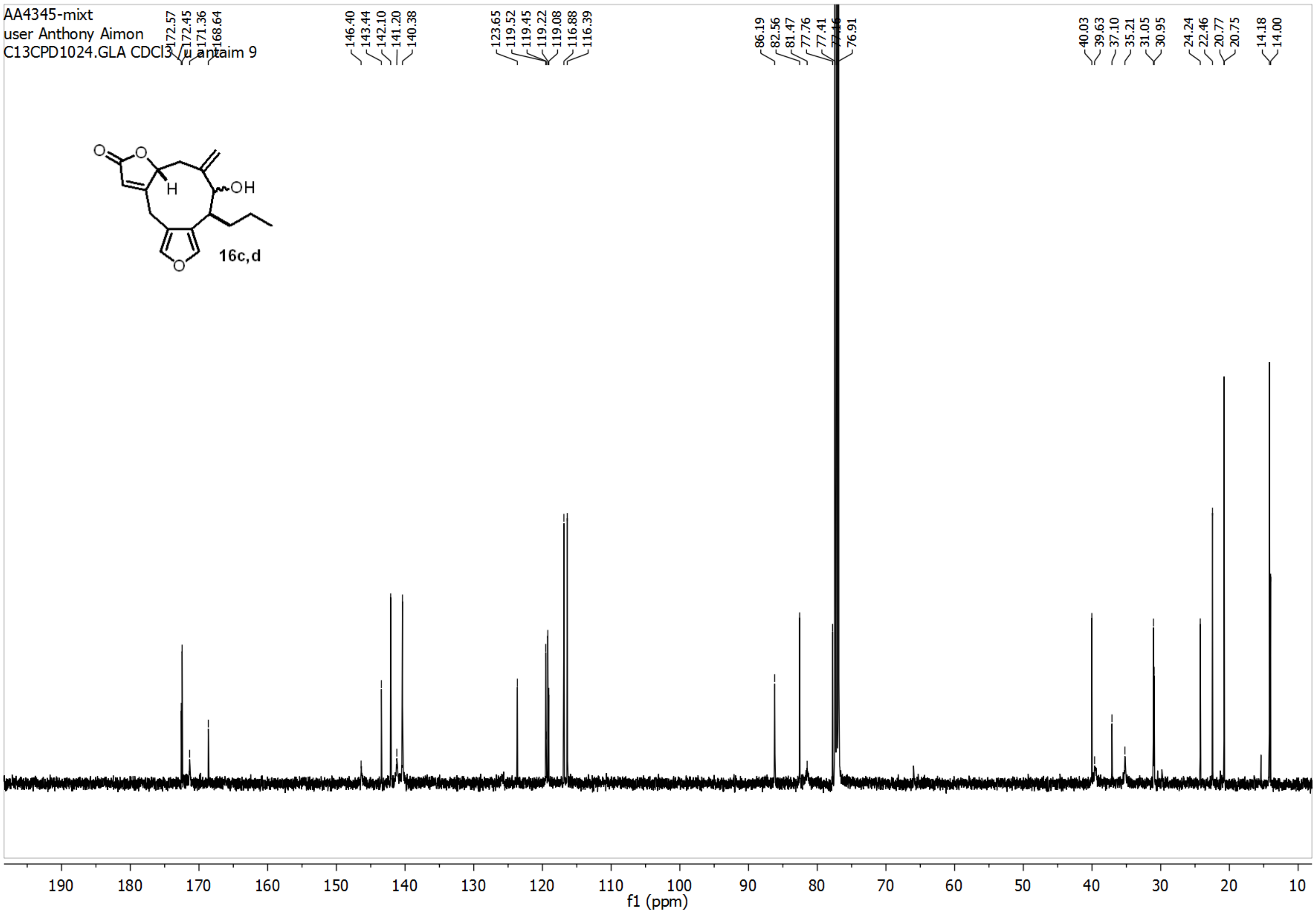
AA4345-mixt
user Anthony Aimon
C13CPD1024.GLA CDCl3 /u antaim 9
172.57
172.45
171.36
168.64
146.40
143.44
142.10
141.20
140.38
123.65
119.52
119.45
119.22
119.08
116.88
116.39
86.19
82.56
81.47
77.76
77.41
76.91
40.03
39.63
37.10
35.21
31.05
30.95
24.24
22.46
20.77
20.75
14.18
14.00
O
O
H
OH
O
16c,d
190
180
170
160
150
140
130
120
110
100
90
80
70
60
50
40
30
20
10
f1 (ppm)

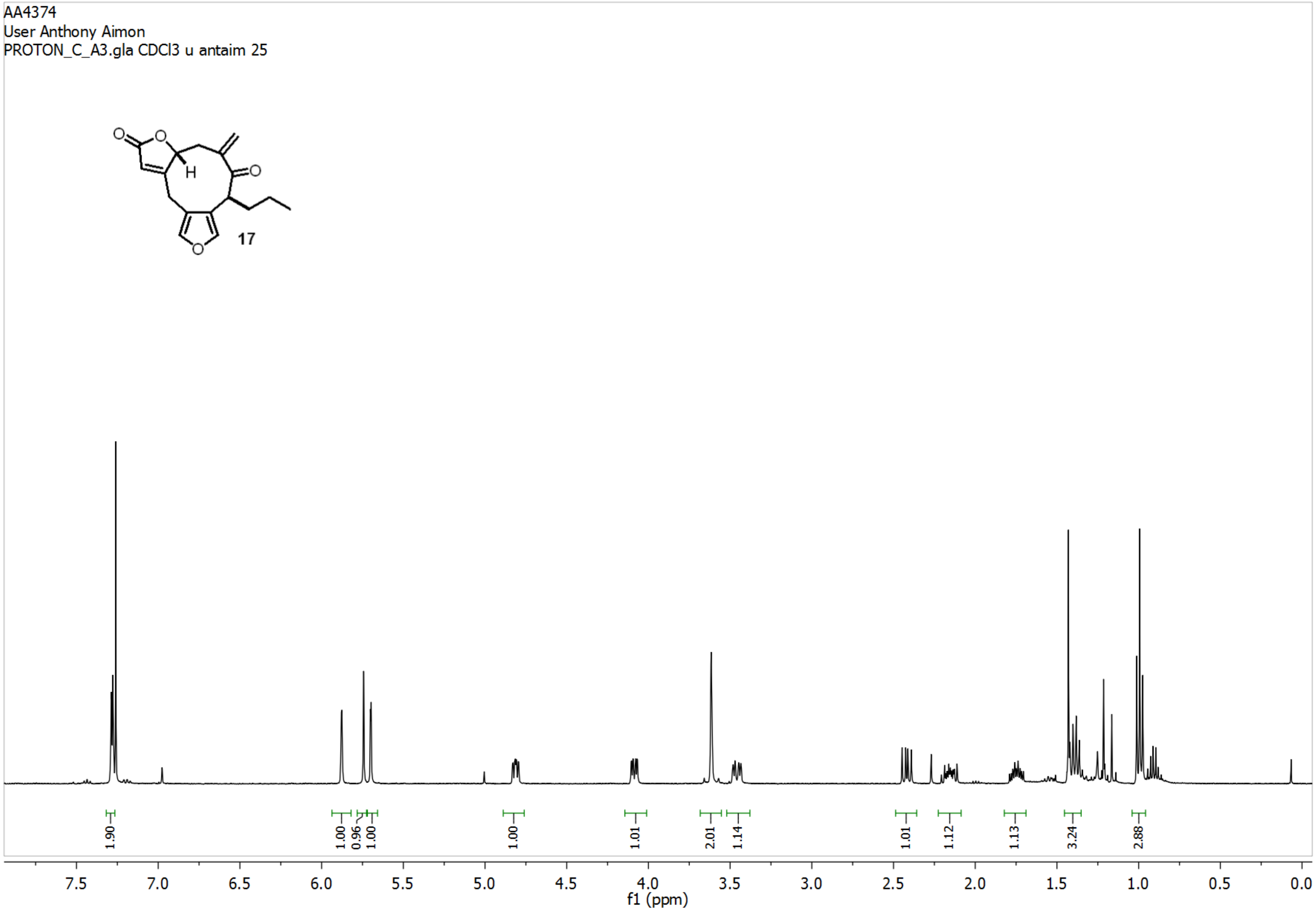
AA4374
User Anthony Aimon
PROTON_C_A3.gla CDCl3 u antaim 25
17
1.90
1.00
0.96
1.00
1.00
1.01
2.01
1.14
1.01
1.12
1.13
3.24
2.88
7.5 7.0 6.5 6.0 5.5 5.0 4.5 4.0 3.5 3.0 2.5 2.0 1.5 1.0 0.5 0.0
f1 (ppm)
